# Magnesium-stabilised transition metal formyl complexes: structures, bonding, and ethenediolate formation†

**DOI:** 10.1039/d2sc02063g

**Published:** 2022-05-16

**Authors:** Joseph M. Parr, Andrew J. P. White, Mark R. Crimmin

**Affiliations:** Molecular Sciences Research Hub, Department of Chemistry, Imperial College London, 82 Wood Lane, White City, Shepherds Bush London W12 0BZ UK m.crimmin@imperial.ac.uk

## Abstract

Herein we report the first comprehensive series of crystallographically characterised transition metal formyl complexes. In these complexes, the formyl ligand is trapped as part of a chelating structure between a transition metal (Cr, Mn, Fe, Co, Rh, W, and Ir) and a magnesium (Mg) cation. Calculations suggest that this bonding mode results in significant oxycarbene-character of the formyl ligand. Further reaction of a heterometallic Cr–Mg formyl complex results in a rare example of C–C coupling and formation of an ethenediolate complex. DFT calculations support a key role for the formyl-intermediate in ethenediolate formation. These results show that well-defined transition metal formyl complexes are potential intermediates in the homologation of carbon monoxide.

## Abstract 2

Herein we report a comprehensive series of crystallographically characterised transition metal formyl complexes.

## Graphical abstract

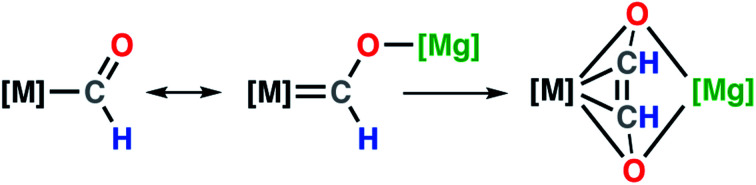

## Introduction

**Scheme 1:**
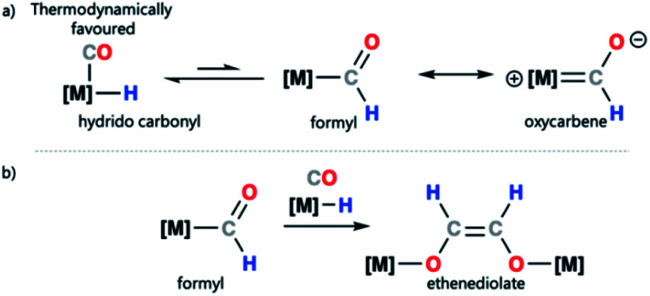
(a) Transition metal–hydride to metal formyl equilibrium and canonical forms of a transition metal formyl complex; (b) proposed ethanediolate formation from a metal formyl complex.

Transition metal formyl complexes are important intermediates in the reduction of carbon monoxide (CO) and carbon dioxide (CO_2_).^1^ For example, transition metal formyl species have been proposed as key intermediates in the reduction of CO with H_2_ to form linear alkanes in the Fischer–Tropsch (F–T) process.^2–13^ Despite their importance, the detailed study of transition metal formyl complexes has been hampered by their low stability. Early studies established that these species undergo facile α-elimination to form the corresponding hydrido carbonyl complexes.^13–25^ The reaction is potentially reversible, but in most cases the hydrido carbonyl is thermodynamically favoured (Scheme 1a).^26–29^ Increased oxycarbene character is expected to increase the metal ligand binding energy through strengthening the metal–carbon bond. This then, in turn, should bias the position of the equilibrium toward the metal formyl species rather than the metal hydrido carbonyl complex.^30–33^

A survey of the Cambridge Crystallographic Data Centre (CCDC) database reveals that despite nearly 50 years of research into transition metal formyl complexes, only a modest number have been structurally characterised (supporting information, Fig. S11 and Table S2†). The paucity of data has meant that there is currently no systematic study into the effect of the transition metal on the bonding of structurally characterised formyl complexes.

Due to the challenge in studying transition metal formyl complexes, even the simplest steps involving these intermediates can be ill-defined. Take for example ethenediolate {C_2_H_2_O_2_}^2−^ formation from combining two CO and two H^−^ ligands on a transition metal (Scheme 1b). This type of C–C bond formation is relevant to F–T type chemistry where metal-bound CO and H^−^ ligands are prevalent.^34–36^ Transition metal formyl complexes were originally proposed as intermediates in ethenediolate formation by Bercaw and co-workers.^37^ Direct support for their involvement is however limited. Studies in the 1980s, largely relied on the use of more stable metal acyl derivatives to explore the fundamental steps of these ligand types.^38,39^ In a single case, Marks and coworkers provided spectroscopic evidence for the involvement of a transient metal formyl intermediate in ethenediolate formation with an actinide complex.^31^ The metal formyl was observed below −50 °C and not isolated. More recently, a number of main group and transition metal systems have been reported for ethenediolate formation,^40–44^ in no case has a metal formyl intermediate been isolated, rather DFT calculations support their involvement in the mechanism for C–C bond formation.

In this study, we document the preparation, structural characterisation, and bonding analysis (NBO, ETS-NOCV, QTAIM) of a complete series of transition metal formyl complexes (M = Cr, Mn, Fe, Co, Rh, W, and Ir). This includes unprecedented examples of crystallographically characterised Cr, Co and Ir formyl complexes. Through reduction of a series of transition metal carbonyl complexes with a molecular magnesium hydride compound, the formyl ligand can be trapped as part of a chelating structure. Further reaction of a chromium formyl complex results in C–C bond formation and an ethenediolate species bridging the chromium and magnesium centres. DFT calculations are consistent with C–C bond formation occurring by stepwise process involving: (i) reduction of the metal formyl to generate an oxymethylene intermediate, (ii) insertion of CO, and (iii) a 1,2-hydride shift. This is an exceedingly rare example in which a well-defined transition metal formyl complex has been shown to be involved in a ethenediolate formation.^34^

## Results and discussion

### Synthesis and characterisation of metal formyl complexes

**Scheme 2:**
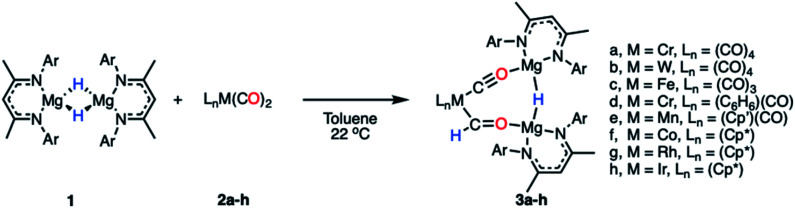
Synthetic procedure for the formation of compounds 3a–h (Ar = 2,6-diisopropylphenyl, Cp′ = methylcyclopentadienyl, Cp* = pentamethylcyclopentadienyl).

Reaction of a magnesium hydride dimer^45^ (1) with a series of transition metal carbonyl complexes (2) in toluene solution furnished the corresponding heterometallic formyl complexes (3) in 21–68% isolated yields (Scheme 2). These reactions occurred readily at 22 °C as evidenced through a marked colour changes and diagnostic spectroscopic data. In C_6_D_6_ solution, the transition metal formyl species 3a–h were characterized by singlet resonances between *δ*_H_ = 13.05–15.11 ppm and *δ*_C_ = 240–310 ppm in the ^1^H and ^13^C{^1^H} NMR spectra respectively. Direct connection between the C and H atoms of the M–CHO fragment was confirmed by heteronuclear single quantum coherence (HSQC) NMR experiments. Further coupling was apparent in cases of spin active transition metals; the formyl ligand of 3b shows ^2^*J*_W–H_ = 9.0 Hz, while 3g shows ^2^*J*_Rh–H_ = 2.1 Hz and ^1^*J*_Rh–C_ = 56.0 Hz. Bridging magnesium hydride resonances ranged *δ*_H_ = 2.86–3.17 ppm in the ^1^H NMR spectra, upfield of the bridging hydride in 1 (*δ*_H_ = 4.02 ppm). No trend between transition metal fragment and hydride resonance was observed.

The infrared data for complexes 3a–h show characteristic stretching frequencies for both the isocarbonyl and carbonyl ligands (Table S3 and Fig. S13–S21†). Terminal carbonyl ligands on 3a–e displayed stretching frequencies at 1916–2067 cm^−1^. 3f–h showed no stretches in this region. The isocarbonyl ligands bridging the magnesium to transition metal centre appear as strong intensity bands at 1674–1791 cm^−1^. Typical formyl stretches appear at a medium intensity in the range 1530–1630 cm^−1^, red-shifted from the carbonyl ligands.^46,47^

Complexes 3a–h show stretching frequencies at 1513–1520 cm^−1^, with the ν(CO) stretch for the M–CHO fragment potentially masked by the C=N stretch of the β-diketiminate ligand. Formyl ligand C–H bonds are observed as a weak intensity stretching frequency at 2546–2635 cm^−1^ for 3a, 3b, 3d, and 3f–h; formyl C–H stretches in 3c and 3e were not observed.

**Fig. 1:**
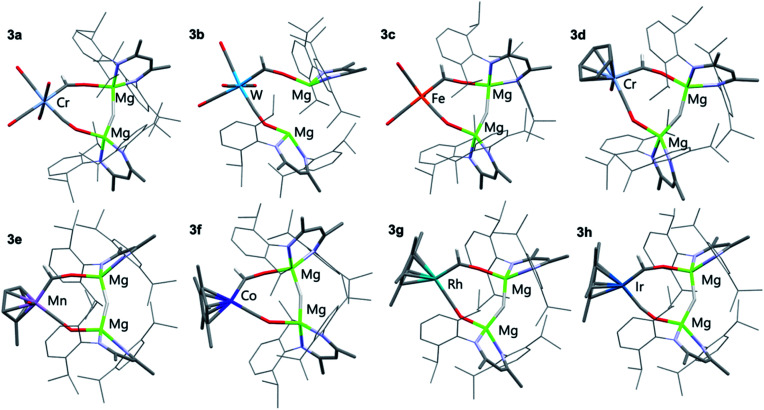
X-ray crystal structures of 3a–h. Solvent molecules and most hydrogen atoms removed for clarity.

All eight members of the series (3a–h) have been characterised in the solid state by single crystal X-ray diffraction analysis (Fig. 1 and Table 1). In all cases these structures showed a key motif involving an 8-membered ring comprised of the transition metal and two magnesium sites, along with the formyl, isocarbonyl and hydride ligands. While all complexes demonstrate the same structural type, only in 3c and 3f is the structure free from disorder, in the other cases positional disorder between the isocarbonyl and formyl sites of the ring is observed.

**Table 1:** Selected structural parameters for M–CHO components for complexes 3

	M–C (XRD)^a^ (Å)	M–C (DFT)^b^ (Å)	C–O (XRD)^a^ (Å)	C–O (DFT)^b^ (Å)	M–C–O (XRD) (°)
3a	2.054(9)	2.048	1.210(7)	1.254	132.0(6)
3b	2.19(2)	2.200	1.26(2)	1.251	130(1)
3c	1.935(9)	1.934	1.221(2)	1.247	128.5(6)
3d	1.95(1)	1.943	1.234(9)	1.263	137.1(8)
3e	1.91(2)	1.903	1.24(1)	1.253	133(1)
3f	1.804(8)	1.816	1.255(9)	1.262	135.9(6)
3g	1.920(6)	1.932	1.270(5)	1.255	134.0(5)
3h	1.80(3)	1.935	1.27(3)	1.260	151(3)

a Average bond length, provided with pooled estimated standard deviation (ESDs) in parentheses where possible.

b Calculated bond length.

Complex 3c, a representative example, crystallises in the *P*1̄ space group, with the five carbon ligands arranged in a trigonal bipyramidal geometry around the iron centre. The formyl ligand occupies the axial position with a Mg–H–Mg bridge to a *cis* equatorial carbonyl ligand. The iron–carbon formyl bond of 1.935(9) Å is markedly longer than the average metal–carbon bonds to the carbonyl ligands 1.81(1) Å.

Within the series 3a–h, M–C bond lengths to the formyl ligand range 1.80(3)–2.19(2) Å. The longest M–C bond lengths are recorded for the most electron deficient d^6^ transition metal fragments (3a and 3b), while some of the shortest M–C distances are recorded for the most electron rich d^8^ transition metal fragments (3f–h). The disorder inherent in these structures results in large estimated standard deviations on the data, but they are also reproduced by Density Functional Theory (DFT) calculations. In most cases, the hydride ligand could be located within the Fourier difference map, while the location of this atom should be treated with caution, the Mg–H–Mg bond angles varied 146(1)–160(3)°. This angle is obtuse, presumably to achieve the geometry for the bimetallic magnesium fragment to bridge two mutually *cis* sites of the transition metal.

### Electronic structure

The electronic structure and bonding interaction between the formyl ligand and transition metal centre were investigated using DFT. Calculations were performed in Gaussian 09 and Orca 4.2.1. Natural Bonding Orbital (NBO) and Extended Transition State-Natural Orbital Chemical Valence (ETS-NOCV) methods were employed to interrogate the bonding interactions in 3a–h. Structures were optimised using the wB97X-D functional, a hybrid basis set was used 6-31G** (C, H), 6-311+G* (N, O) while the SDDAll pseudopotential and associated basis sets were used to describe Mg and M centres.^48–53^

NBO calculations were performed on complexes 3a–h. Analysis of the Natural Population Analysis (NPA) charges suggests that the binding interaction between the transition metal fragment and dimagnesium hydride fragment is largely ionic. The hydride (−0.8) and oxygen atoms (−0.9) directly attached to magnesium bear significant negative charge while these magnesium sites themselves are highly electropositive (+1.8). Comparison of data for 3a and 3b to the theoretical models [M(CO)_5_(CHO)]^−^ (M = Cr, Mo, W) shows little change in the NPA charges, supporting the idea that there is a large ionic contribution to the bonding between the transition metal and main group fragments. QTAIM calculations point to the same conclusion, with hydride (−0.8), oxygen (−1.4) and magnesium (+1.8) charges complimenting the NBO data (Tables S5, S6 and S9†).

Comparison of both the NPA charges and Wiberg bond indices (WBI) for selected sets of complexes within the series allows the influence of d^*n*^ electron count, ligand, and transition metal on the bonding to be speculated upon (Table 2). The trends in the calculated data are such that only a course comparison can be made, however comparing examples based on electron-deficient group 6 and 8 metal fragments (*e.g.*3a–c) with more electron-rich group 9 metals (*e.g.*3f–h) suggests that the oxycarbene character increases with increasing electron-density on the metal. This proposed trend is manifest in higher M–C WBIs, lower C–O WBIs, decreased charge on M, and increased charge on O for the more-electron rich members of the series.

**Table 2:** Select Natural Population Analysis (NPA) charges and Wiberg Bond Indices (WBI) for complexes 3a–h. Complete NBO data can be found in the ESI (Tables S5 and S6)

	NPA charge					WBI		
	M	C	O	Mg	H	M–C	C–O	O–Mg
3a	−1.29	0.27	−0.86	1.78	−0.80	0.55	1.52	0.052
3b	−0.86	0.19	−0.86	1.76	−0.80	0.60	1.53	0.054
3c	−0.46	0.25	−0.85	1.77	−0.80	0.57	1.52	0.055
3d	−0.66	0.30	−0.91	1.77	−0.80	0.87	1.44	0.057
3e	−0.42	0.31	−0.87	1.77	−0.80	0.77	1.50	0.06
3f	0.21	0.21	−0.91	1.77	−0.79	0.79	1.42	0.06
3g	0.17	0.23	−0.89	1.77	−0.79	0.85	1.44	0.058
3h	0.28	0.18	−0.90	1.77	−0.79	0.95	1.40	0.058

ETS-NOCV calculations were performed on complexes 3a–h to obtain quantitative data on the nature and strength of the metal–ligand interaction (Tables S7 and S8†). Before embarking on this analysis, it is useful to consider the frontier molecular orbitals of a bent {CHO}^−^ triatomic; the HOMO shows significant carbon based lone-pair character, while the LUMO is a π* orbital with the largest coefficient on the carbon atom (Fig. S31†). In all cases for 3a–h the principal contribution (∇*ρ*_1_) to the bonding interaction is the σ-donation of the HOMO of the {CHO}^−^ ligand to a transition-metal d-orbital. The secondary (∇*ρ*_2_) and tertiary (∇*ρ*_3_) interactions involve π back-donation from a metal-based orbital to the orthogonal π*-orbitals of the {CHO}^−^ ligand.‡‡For Ir, further splitting of the metal-based d-orbitals (due to overlap with ligand SALCs of CO) results in both *ρ*_1_ and *ρ*_2_ interactions being σ-donation from the HOMO of {CHO}^−^ to empty orbitals on the Ir fragment. *ρ*_3_ is π-backdonation.

**Fig. 2:**
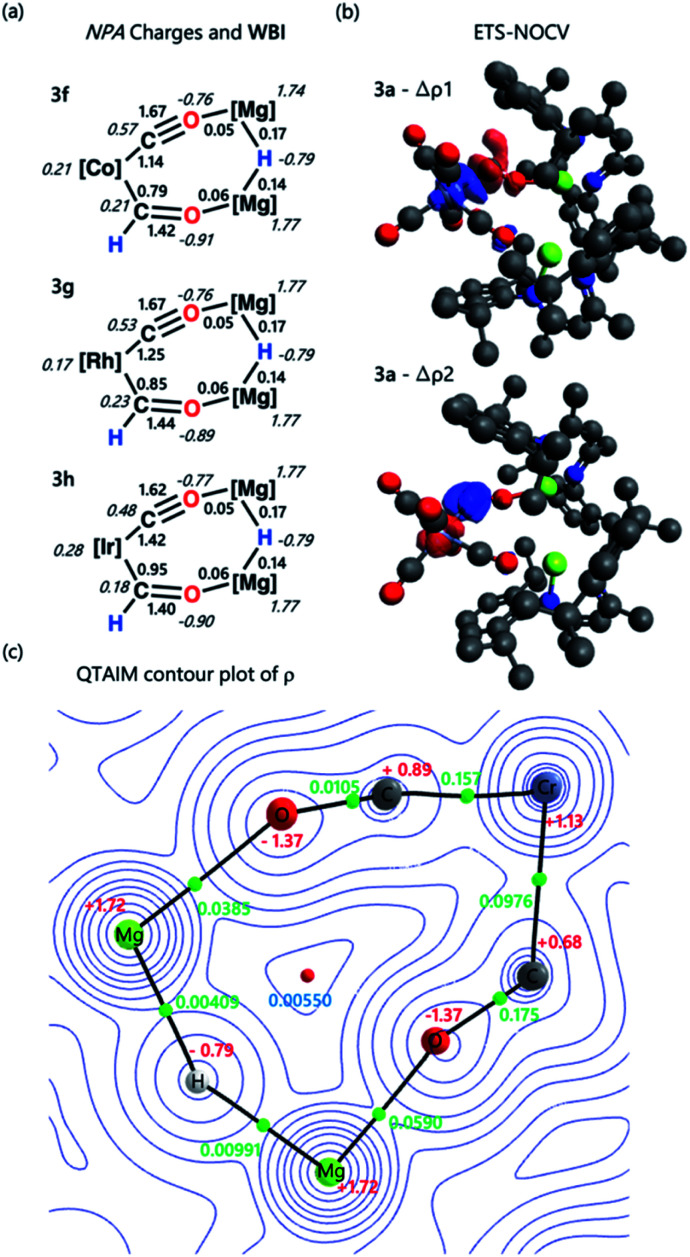
(a) Select NPA charges and Wiberg bond indices for complexes 3f–h; (b) select ETS-NOCV deformation density data for complex 3a. Charge flow is from red to blue; (c) QTAIM contour plot of *ρ* for 3a. Charges are depicted in red, electron density at the bond critical point (*ρ*) in green, and ring critical point in blue. Complete NBO, ETS-NOCV, and QTAIM data can be found in the ESI.†

These donor–acceptor interactions can be visualised in the deformation density plots (Fig. 2b and Tables S11–S13†). The total orbital interaction between the formyl ligand and transition metal fragment, Δ*E*_orb_, broadly increases across the first–row transition metals (3a*vs.*3e; 3d*vs.*3f) and down the triad (3f*vs.*3g*vs.*3h). Data that could again be interpreted in terms of increased oxycarbene character increasing the strength of the bonding interaction with more electron-rich transition metal fragments.§§While the treatment allows analysis of the key bonding interaction between the formyl ligand and the transition metal, it does not lead to the lowest total orbital interaction energies Δ*E*_ORB_. A second set of ETS-NOCV calculations split complexes 3 into anionic and cationic fragments by dividing at the Mg–O bond, giving consistently lower Δ*E*_ORB_ values (Table S8 and Fig. S34†).

### Solution stability and ethenediolate formation

To further assess their stability in solution, half-lives for complexes of 3a–h in C_6_D_6_ at 22 °C have been measured (Table S4 and Fig. S22–S30†). In all cases kinetics followed a first-order decay, consistent with an intramolecular pathway: 3a (*t*_1/2_ = 2 h), 3b (*t*_1/2_ = 2 h), 3c (*t*_1/2_ = 57 h), 3d (*t*_1/2_ = 136 h), 3e (*t*_1/2_ = 224 h), 3f (*t*_1/2_ = 330 h), 3g (*t*_1/2_ = 217 h), 3h (*t*_1/2_ = 533 h). The most stable complexes in this series 3d–h are those which contain electron-rich and sterically demanding ligands on the transition metal fragment. It has been previously suggested that transition metal formyl complexes can be stabilised by inclusion of a sterically demanding ligand, commonly a bulky phosphine.^3,4,30,54,55^ The half-life for complex 3a (*t*_1/2_ = 2 h), was identical in C_6_D_6_ and THF solvent and did not change when running the reaction under 1 atm. of CO. These results suggest that neither the solvent nor the concentration of CO in solution influences the stability of the formyl ligand.

**Scheme 3:**
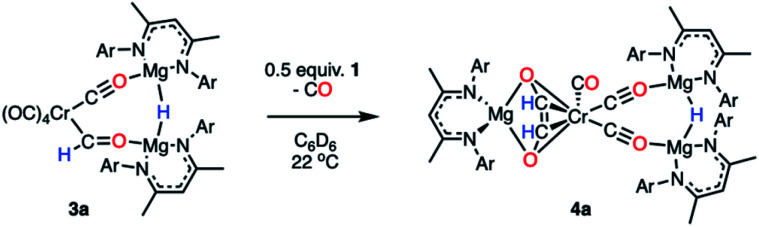
Transformation of 3a to 4a, a C–C coupled product.

**Fig. 3:**
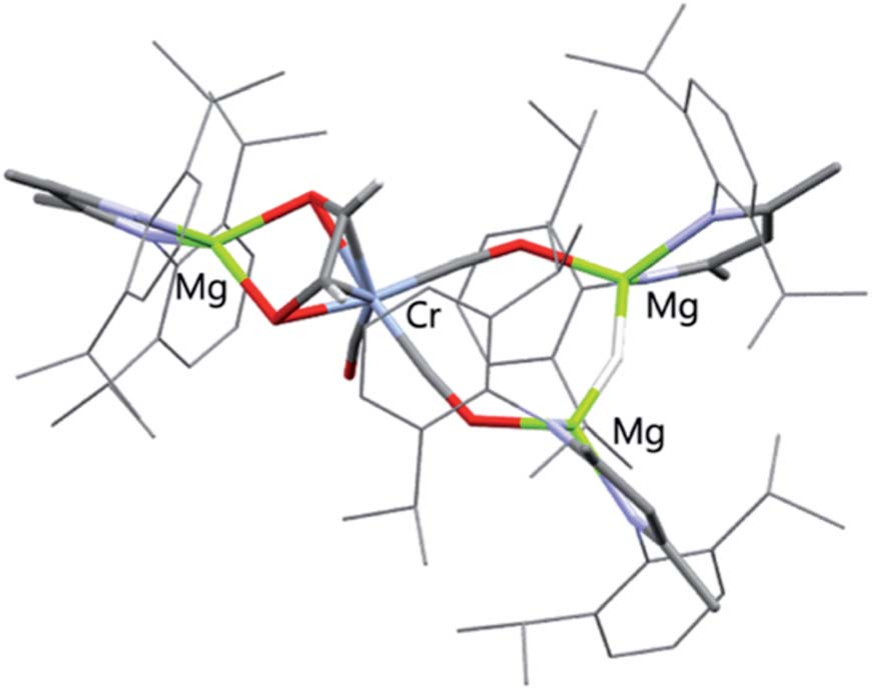
X-ray crystal structures of 4a. Solvent molecules and most hydrogen atoms removed for clarity.

Onward reaction of 3a yielded a single well-defined product 4a (Scheme 3). 4a can be synthesised *via* three routes: thermolysis of 3a, reaction of 3a with 0.5 equiv. of 1, and direct 1 : 1 reaction of 1 with 2a overnight; yields ranged 62–70% (NMR), 31–51% (isolated crystals). 4a has been characterised by multinuclear NMR spectroscopy and single crystal X-ray diffraction (Fig. 3). The CO coupling pathway involving a well-defined formyl intermediate stands in stark contrast to the expected α-elimination reaction. 4a comprises an ethenediolate {C_2_H_2_O_2_}^2−^ unit bridging chromium and magnesium centres. From the perspective of chromium, the new ligand is reminiscent of a 6-electron (2p + 2 oxygen LP) analogue of a butadiene motif.

In solution, HSQC experiments on 4a led to the assignment of the ethenediolate protons as two singlet resonances at *δ*_H_ = 4.33 (1H) and 5.45 ppm (1H), associated carbon resonances occur at *δ*_C_ = 110.2 and 110.5 ppm. The spectroscopic data are consistent with that reported for related magnesium ethenediolate complexes by Hill and Jones.^43,44^ The hydride bridging the two magnesium centres appears as a singlet resonance at *δ*_H_ = 2.72 ppm. In the solid state, the key ethenediolate ligand spans chromium and magnesium centre. Respective C=C and C–Cr bond lengths of 1.369(5) and 2.104(3)–2.109(3) Å indicate an ligand binding in a η^4^-fashion to the central chromium atom. The {C_2_H_2_O_2_}^2−^ unit coordinates through oxygen to a magnesium centre with Mg–O bond lengths of 1.986(2) and 1.992(2) Å.

**Fig. 4:**
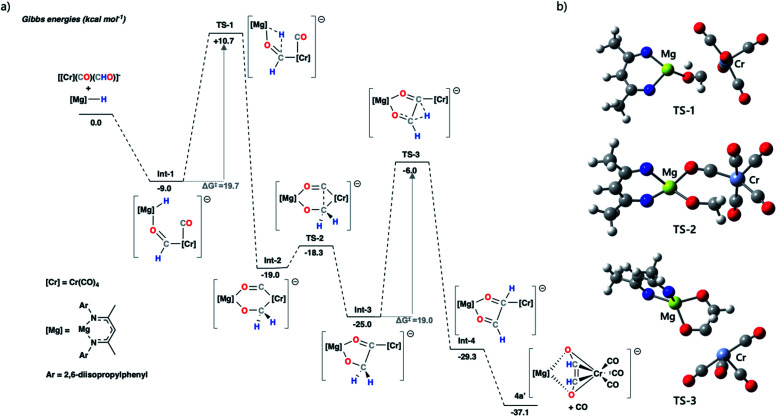
(a) Calculated potential energy surface for ethenediolate formation from a model chromium formyl anion. The structures were optimised using the ωB97X-D functional, a hybrid basis set was used 6-31G** (C, H), 6-311+G* (N, O) while the SDDAll pseudopotential and associated basis sets were used to describe Mg and Cr centres. Solvent corrections were applied *via* single point corrections, using the PCM (solvent = toluene) model; (b) optimised geometry structures for transition states TS-1, TS-2 and TS-3; 2,6-diisopropylphenyl and most hydrogen atoms removed for clarity (carbon = grey, chromium = teal, hydrogen = white, magnesium = yellow, nitrogen = blue, oxygen = red).

DFT calculations were used to investigate ethenediolate formation from a formyl intermediate. Calculations were performed on a model system comprised of [Cr(CO)_5_(CHO)]^−^ and monomeric 1. Such a simplification is necessary for computational cost, however this system still provides insight into the most likely steps involved in the mechanism for C–C bond formation. The overall formation of the ethenediolate from the chromium formyl complex is exergonic with 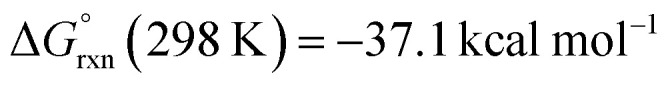. The most plausible pathway calculated for ethenediolate formation follows that originally postulated by Bercaw and co-workers (Fig. 4).^37^ Importantly, our work now connects a well-defined and structurally characterised formyl intermediate to an ethenediolate product.

The stepwise mechanism is initiated by the coordination of 1 to the oxygen atom of the formyl ligand of [Cr(CO)_5_(CHO)]^−^ to give Int-1. Int-1 undergoes hydride-transfer from Mg to Cr–CHO *via*TS-1 (Δ*G*^‡^ = 19.7 kcal mol^−1^), forming Int-2. This step is formally a hydromagnesiation of the transition metal formyl and generates a new oxymethylene ligand. From Int-2, C–C coupling proceeds through a migratory insertion reaction that is characterised by a low energy transition state TS-2 (Δ*G*^‡^ = 0.7 kcal mol^−1^). TS-2 proceeds to a five-coordinate chromium complex Int-3. Int-3 can then undergo a 1,2-H shift *via*TS-3 (Δ*G*^‡^ = 19.0 kcal mol^−1^) to form the ethenediolate ligand which at this point is coordinated to chromium in an η^1^-fashion. Subsequent dissociation of CO gives the product 4a′ and occurs with translation of the ethenediolate to an η^4^-mode.

NBO analysis was performed on the key transition states on the pathway. Analysis of the hydromagnesiation step shows an increased positive charge on the H atom as it moves from Mg in Int-1 (−0.72) toward C in TS-1 (−0.51). At the same time there is an expected decrease in the WBI for the Mg–H bond (0.40 to 0.15; Int-1 to TS1). The 1,2-H atom shift occurs with re-organisation of the electron density in the {C_2_H_2_O_2_}^2−^ fragment. Most notably, from Int-3 to TS3 the C–C bond WBI increases from 0.95 to 1.24 as the ethenediolate forms (Fig. S43†). Alternative mechanistic pathways gave considerably higher energy barriers or did not proceed towards the known ethenediolate product (Fig. S44†). For example, the direct hydromagnesiation of a carbonyl ligand of [Cr(CO)_5_(CHO)]^−^ with 1 to form a bis(formyl) intermediate was found to proceed by activation barrier of over 50 kcal mol^−1^, effectively ruling out this pathway.

## Conclusions

In summary, we report the first comprehensive series of transition-metal formyl complexes (M = Cr, Mn, Fe, Co, Rh, W, Ir). These include unprecedented examples of crystallographically characterised Cr, Co and Ir formyl complexes. In all cases, the metal bound formyl ligand is stabilised by a heterometallic effect. We investigated this oxycarbene electronic structure through a series of density functional theory calculations (NBO, ETS-NOCV, QTAIM), and concluded that more electron rich metal centres likely result in more oxycarbene character, and thus stabilisation of the metal-formyl ligand. Remarkably, onwards reaction of a chromium formyl complex yields C–C coupling and an isolable ethenediolate complex. This serves as the first direct example of carbon–carbon bond formation at a well-defined and isolable metal-formyl complex.

## Data availability

Experimental procedures, details of the calculations, and additional data can be found in the ESI† (.pdf). X-ray data is available in .cif format.

## Author contributions

JMP conducted all experimental and computational work. AJPW and JMP solved and refined single crystal XRD data. All authors contributed to writing the manuscript.

## Conflicts of interest

There are no conflicts to declare.

## Supplementary Material

SC-013-D2SC02063G-s001

SC-013-D2SC02063G-s002
