# Supplementary material for: Magnesium-stabilised transition metal formyl complexes: structures, bonding, and ethenediolate formation
Source: Chem Sci. 2022 May 16;13(22):6592–8. doi: 10.1039/d2sc02063g (PMC9172563; doi:10.1039/d2sc02063g)
Supplement: SC-013-D2SC02063G-s001 [file SC-013-D2SC02063G-s001.pdf]

Electronic Supplementary Material (ESI) for Chemical Science.
This journal is © The Royal Society of Chemistry 2022

Magnesium-Stabilized Transition Metal Formyl Complexes: Structures, Bonding, and Ethenediolate Formation

Joseph M. Parr,[a] Andrew J. P. White,[a] and Mark R Crimmin*[a]

Corresponding author. Email: m.crimmin@imperial.ac.uk

[a] Molecular Sciences Research Hub, Department of Chemistry, Imperial College London, 82 Wood Lane, White City, Shepherds Bush, London, W12 0BZ, UK.

Contents

1. General Experimental……………………………………………………………….…......1
2. Synthetic Procedures……………………………………………….………………………2
3. X-ray Crystallography…………………………………………………...……………….12
4. IR Spectroscopy…………………………………….……………………………………..28
5. Stability Studies…………………………………………………………………………..34
6. Computational Methods…………………………………………………………………..40
7. References………………………………………………………………………………...62

1. General Experimental

All manipulations were carried out under standard Schlenk-line or glovebox techniques under an inert atmosphere of nitrogen. A MBraun Labmaster glovebox was employed operating at concentrations of H_2O and O_2 below 0.1 ppm. Glassware was dried for 12 hours at 120 °C prior to use. C_6D_6 was freeze-pump thaw degassed thrice and stored over molecular sieves for twelve hours before use. All other solvents were dried using a Grubbs type solvent purification system. ^{1}H, ^{13}C and HSQC NMR experiments were run within J-Young Tap NMR tubes on 400 MHz BRUKER machines. Spectra were referenced to known residual solvent peaks. NMR analysis was conducted in MestReNova with baseline and phase corrections applied to spectra. Chemical shift values are reported in ppm and coupling constants *J* in Hz. Infrared spectra were obtained on a Cary630 spectrometer (placed with in an MBraun glovebox) from crystalline solids or toluene thin-films on an ATR cell. Microanalysis (CHN) were performed under inert atmosphere by Elemental Microanalysis Ltd. **1**[1] and **2f/h**[2] were prepared as described previously. Reagent grade carbon monoxide (99.97 %) was purchased from BOC Ltd and used as received. All other reagents were purchased from commercial vendors and used without further purification.

2. Synthetic Procedures

Synthesis of **3a**

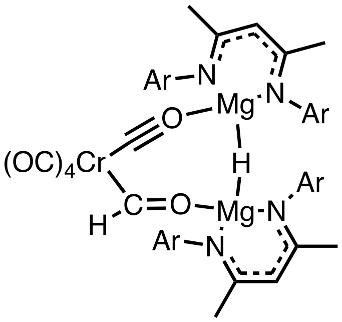

In a N_2 filled glovebox, a suspension of **1** (100 mg, 0.113 mmol) in toluene (3 mL) was added to a solution of [$Cr(CO)_6$] (24.9 mg, 0.113 mmol) in toluene (2 mL) and the resulting yellow solution stirred at 25 °C for 10 minutes. The solution was dried under vacuum, the crude product was extracted with *n*-hexane (3 x 1 mL) and filtered through a glass fibre. Recrystallisation from a toluene/*n*-hexane (1:1 v:v) mixture at -35 °C yielded yellow block crystals suitable for x-ray diffraction studies. The mother liquor was decanted, and the crystals washed with *n*-pentane (3 x 1 mL) and dried under vacuum to yield **3a** as yellow blocks (34 mg, 0.031mmol, 27 %).

^{1}H NMR (400 MHz, C_6D_6) δ: 0.29 (broad d, 6H, CH(C**H**$_3$)$_2$), 0.86-1.38 (overlapping m, 42H, CH(C**H**$_3$)$_2$), 1.50-1.56 (overlapping s, 12H, NC(C**H**$_3$)), 2.85 (sept, $^3J_{H\text{-}H}$ = 6.2 Hz, 2H, C**H**(CH$_3$)$_2$), 3.03 (s, 1H, Mg-**H**-Mg), 3.11 (m, 2H, C**H**(CH$_3$)$_2$), 3.24 (m, 2H, C**H**(CH$_3$)$_2$), 3.45 (sept, $^3J_{H\text{-}H}$ = 6.3 Hz, 2H, C**H**(CH$_3$)$_2$), 4.81 (s, 2H, (CH$_3$)C(C**H**)C(CH$_3$)), 7.00-7.20 (12H, Ar-**H**), 13.56 (s, 1H, Cr-C**H**O).

Due to the thermal instability of **3a** in solution complete ^{13}C NMR data could not be collected. Selected resonances were obtained from a combination of ^{13}C{^{1}H} NMR and HSQC experiments. ^{13}C NMR (101 MHz, C_6D_6) δ: 96.0 (2x (**C**H)C(CH$_3$)$_2$), 169.1 (N**C**(CH$_3$)), 169.3 (N**C**(CH$_3$)), 169.4 (N**C**(CH$_3$)), 169.9 (N**C**(CH$_3$)), 223.6 (Cr-**C**O-Mg), 345.3 (Cr-**C**HO).

IR (ATR, cm^{-1}), ν_{CO}: 1776 (s, CrCOMg), 1943 (s, CrCO), 1947 (m, CrCO), 1987 (s, CrCO); ν_{CH}: 2590 (w, CrCHO).

Anal. Calc. ($CrC_{64}H_{84}N_4O_6Mg_2$): C, 69.50; H, 7.66; N, 5.07. Found: C, 70.65; H, 8.56; N, 5.09.

Synthesis of **3b**

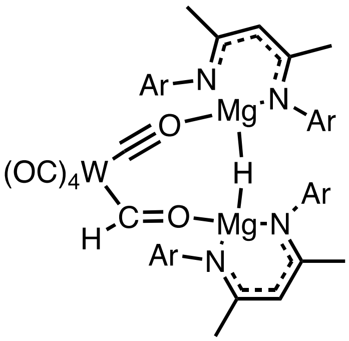

In a N_2 filled glovebox, a suspension of **1** (100 mg, 0.113 mmol) in toluene (3 mL) was added to a solution of [W(CO)$_6$] (39.8 mg, 0.113 mmol) in toluene (2 mL) and the resulting yellow solution stirred at 25 °C for 10 minutes. The solution was dried under vacuum, the crude product was extracted with *n*-hexane (3 x 1 mL) and filtered through a glass fibre. Recrystallisation from a toluene/*n*-hexane (1:1 v:v) mixture at -35 °C yielded yellow block crystals suitable for x-ray diffraction studies. The mother liquor was decanted, and the crystals washed with *n*-pentane (3 x 1 mL) and dried under vacuum to yield **3b** as yellow blocks (50 mg, 0.040 mmol, 36 %).

^{1}H NMR (400 MHz, C_6D_6) δ: 0.21 (broad d, 3H, CH(C**H**$_3$)$_2$), 0.40 (broad d, 3H, CH(C**H**$_3$)$_2$), 0.86-1.39 (overlapping m, 42H, CH(C**H**$_3$)$_2$), 1.50-1.60 (overlapping s, 12H, NC(C**H**$_3$)), 2.86 (sept, $^3J_{H\text{-}H}$ = 6.1 Hz, 2H, C**H**(CH$_3$)$_2$), 3.10 (s, 1H, Mg-**H**-Mg), 3.12-3.28 (overlapping m, 4H, C**H**(CH$_3$)$_2$), 3.43 (sept, $^3J_{H\text{-}H}$ = 6.1 Hz, 2H, C**H**(CH$_3$)$_2$), 4.81 (s, 2H, (CH$_3$)C(C**H**)C(CH$_3$)), 6.99-7.19 (12H, Ar-**H**), 14.80 (s, $^2J_{W\text{-}H}$ = 9.0 Hz, 1H, W-C**H**O).

^{13}C NMR (101 MHz, C_6D_6) δ 24.1 (4x NC(**C**H$_3$)), 24.3 (CH(**C**H$_3$)$_2$), 24.4 (2x CH(**C**H$_3$)$_2$), 24.5 (2x CH(**C**H$_3$)$_2$), 24.6 (CH(**C**H$_3$)$_2$), 24.8 (2x CH(**C**H$_3$)$_2$), 25.0 (CH(**C**H$_3$)$_2$), 27.0 (CH(**C**H$_3$)$_2$), 28.1 (CH(**C**H$_3$)$_2$), 28.3 (CH(**C**H$_3$)$_2$), 28.4 (CH(**C**H$_3$)$_2$), 28.6 (CH(**C**H$_3$)$_2$), 28.8 (2x CH(**C**H$_3$)$_2$), 96.1 (2x (**C**H)C(CH$_3$)$_2$), 124.0 (Ar-**C**), 124.1 (Ar-**C**), 124.2-124.6 (overlapping Ar-**C**), 125.4 (Ar-**C**), 125.5 (Ar-**C**), 126.0 (Ar-**C**), 142.5 (Ar-**C**), 142.6 (Ar-**C**), 142.8 (Ar-**C**), 142.9 (Ar-**C**), 143.0 (Ar-**C**), 143.3 (Ar-**C**), 146.7 (Ar-**C**), 169.1 (2x N**C**(CH$_3$)), 169.2 (N**C**(CH$_3$)), 169.4 (N**C**(CH$_3$)), 191.1 (s, $^1J_{W\text{-}C}$ = 63.3 Hz = W-**C**O-Mg), 323.3 (s, W-**C**HO). *Quaternary ^{13}C resonance of the **C**H(CH$_3$)$_2$ and **C**$_{ipso}$ atoms on the 2,6-diisopropylphenyl unit not observed.*

IR (ATR, cm^{-1}), ν_{CO}: 1769 (s, WCOMg), 1936 (s, WCO), 1942 (s, WCO), 1987 (w, WCO), 2067 (w, WCO); ν_{CH}: 2594 (w, WCHO).

Anal. Calc. ($WC_{64}H_{84}N_4O_6Mg_2$): C, 62.10; H, 6.84; N, 4.53. Found: C, 61.93; H, 6.98; N, 4.23.

Synthesis of **3**c

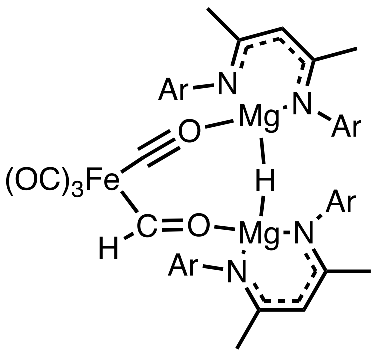

In a N_2 filled glovebox, a suspension of **1** (100 mg, 0.113 mmol) in toluene (3 mL) was added to a solution of [$Fe(CO)_5$] (15.2 µL, 0.113 mmol) in toluene (2 mL) and the resulting yellow solution stirred at 25 °C for 10 minutes. The solution was dried under vacuum, the crude product was extracted with *n*-hexane (3 x 1 mL) and filtered through a glass fibre. Recrystallisation from a toluene/*n*-hexane (1:1 v:v) mixture at -35 °C yielded yellow block crystals suitable for x-ray diffraction studies. The mother liquor was decanted, and the crystals washed with *n*-pentane (3 x 1 mL) and dried under vacuum to yield **3c** as yellow blocks (75 mg, 0.068 mmol, 61 %).

^{1}H NMR (400 MHz, C_6D_6) δ: 0.36 (d, $^3J_{H-H}$ = 6.8 Hz, 3H, CH(C**H**$_3$)$_2$), 0.48 (d, 3H, CH(C**H**$_3$)$_2$), 0.88 (multiplet, 3H, CH(C**H**$_3$)$_2$), 1.03 (d, $^3J_{H-H}$ = 6.8 Hz, 3H, CH(C**H**$_3$)$_2$), 1.07-1.41 (overlapping multiplets, 36H, CH(C**H**$_3$)$_2$), 1.51-1.63 (overlapping s, 12H, NC(C**H**$_3$)), 2.70 (sept, $^3J_{H-H}$ = 6.7 Hz, 1H, C**H**(CH$_3$)$_2$), 2.83 (sept, $^3J_{H-H}$ = 6.8 Hz, 1H, C**H**(CH$_3$)$_2$), 2.86 (s, 1H, Mg-**H**-Mg), 3.03-3.17 (multiplet, 3H, C**H**(CH$_3$)$_2$), 3.27 (sept, $^3J_{H-H}$ = 6.7 Hz, 1H, C**H**(CH$_3$)$_2$), 3.42 (sept, $^3J_{H-H}$ = 6.9 Hz, 1H, C**H**(CH$_3$)$_2$), 3.51 (sept, $^3J_{H-H}$ = 6.9 Hz, 1H, C**H**(CH$_3$)$_2$), 4.80 (s, 1H, (CH$_3$)C(C**H**)C(CH$_3$)), 4.82 (s, 1H, (CH$_3$)C(C**H**)C(CH$_3$)), 7.00-7.22 (12H, Ar-**H**), 13.30 (s, 1H, Fe-C**H**O).

^{13}C NMR (101 MHz, C_6D_6) δ 20.5 (2x NC(**C**H$_3$)), 20.8 (2x NC(**C**H$_3$)), 23.1 (CH(**C**H$_3$)$_2$), 23.2 (CH(**C**H$_3$)$_2$), 23.3 (CH(**C**H$_3$)$_2$), 23.5 (CH(**C**H$_3$)$_2$), 23.8 (CH(**C**H$_3$)$_2$), 24.0 (CH(**C**H$_3$)$_2$), 24.2 (CH(**C**H$_3$)$_2$), 24.5 (CH(**C**H$_3$)$_2$), 24.6(CH(**C**H$_3$)$_2$), 24.7 (CH(**C**H$_3$)$_2$), 25.0 (CH(**C**H$_3$)$_2$), 25.2 (CH(**C**H$_3$)$_2$), 26.5 (CH(**C**H$_3$)$_2$), 27.0 (CH(**C**H$_3$)$_2$), 27.8 (CH(**C**H$_3$)$_2$), 27.9 (CH(**C**H$_3$)$_2$), 28.1 (**C**H(CH$_3$)$_2$), 28.4 (**C**H(CH$_3$)$_2$), 28.7 (**C**H(CH$_3$)$_2$), 28.8 (**C**H(CH$_3$)$_2$), 94.3 ((CH)C(CH$_3$)$_2$), 95.7 ((**C**H)C(CH$_3$)$_2$), 123.4 (Ar-**C**), 123.6 (Ar-**C**), 124.0 (Ar-**C**), 124.3 (Ar-**C**), 124.5 (Ar-**C**), 124.6 (Ar-**C**), 124.7 (Ar-**C**), 125.6 (Ar-**C**), 126.0 (Ar-**C**), 126.1 (Ar-**C**), 136.4 (Ar-**C**), 141.3 (Ar-**C**), 141.6 (Ar-**C**), 142.1 (Ar-**C**), 142.4 (Ar-**C**), 142.7 (Ar-**C**), 142.8 (Ar-**C**), 143.1 (Ar-**C**), 143.3 (Ar-**C**), 143.7 (Ar-**C**), 144.5 (Ar-**C**), 144.6 (Ar-**C**), 145.2 (Ar-**C**), 146.9 (Ar-**C**), 167.6 (N**C**(CH$_3$)), 169.0 (N**C**(CH$_3$)), 169.6 (N**C**(CH$_3$)), 170.4 (N**C**(CH$_3$)), 213.3 (Fe-**C**O), 235.0 (Fe-**C**O-Mg), 307.2 (Fe-**C**HO).

IR (ATR, cm^{-1}), ν_{CO}: 1784 (s, FeCOMg), 1903 (s, FeCO), 1951 (s, FeCO), 1981 (w, FeCO).

Anal. Calc. ($FeC_{63}H_{84}N_4O_5Mg_2$): C, 69.94; H, 7.83; N, 5.18. Found: C, 64.90; H, 7.47; N, 4.61. Accurate CHN analysis could not be obtained due to the instability of this complex.

Synthesis of **3d**

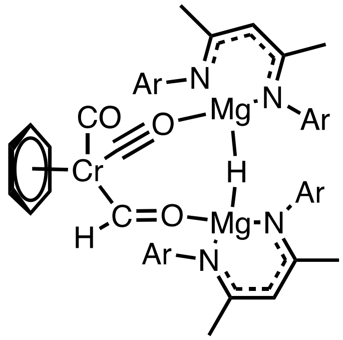

In a N_2 filled glovebox, a suspension of **1** (100 mg, 0.113 mmol) in toluene (3 mL) was added to a solution of [Cr(η^6-C_6H_6)$(CO)_3$] (24.2 mg, 0.113 mmol) in toluene (2 mL) and the resulting orange solution stirred at 25 °C for 10 minutes. The solution was dried under vacuum, the crude product was extracted with *n*-hexane (3 x 1 mL) and filtered through a glass fibre. Recrystallisation from a toluene/*n*-hexane (v:v 1:1) mixture at -35 °C yielded orange crystals suitable for x-ray diffraction studies. The mother liquor was decanted, and the crystals washed with *n*-pentane (3 x 1 mL) and dried under vacuum to give **3d** as orange crystals (69 mg, 0.059 mmol, 53 %).

^{1}H NMR (400 MHz, C_6D_6) δ: 0.28-0.44 (m, 6H, CH(C**H**$_3$)$_2$), 0.89 (m, 6H, CH(C**H**$_3$)$_2$), 1.14-1.34 (overlapping m, 30H, CH(C**H**$_3$)$_2$), 1.40 (d, $^3J_{H\text{-}H}$ = 6.8 Hz, 6H, CH(C**H**$_3$)$_2$), 1.55 (s, 3H, NC(C**H**$_3$)), 1.56 (s, 3H, NC(C**H**$_3$)), 1.63 (s, 3H, NC(C**H**$_3$)), 1.65 (s, 3H, NC(C**H**$_3$)), 2.94 (sept, $^3J_{H\text{-}H}$ = 6.3 Hz, 2H, (CH_3)C**H**(CH_3)), 3.16 (s, 1H, Mg-H-Mg), 3.33 (sept, $^3J_{H\text{-}H}$ = 6.8 Hz, 4H, C**H**(CH_3)$_2$), 3.53 (broad s, 2H, C**H**(CH_3)$_2$), 4.84 (s, 2H, (CH_3)C(C**H**)C(CH_3)), 4.32 (s, 6H, C_6**H**$_6$), 7.03-7.24 (12H, Ar-**H**), 15.11 (s, 1H, Cr-C**H**O).

^{13}C NMR (101 MHz, C_6D_6) δ: 22.3 (2x NC(**C**H$_3$)), 23.1 (2x NC(**C**H$_3$)), 23.8 (CH(**C**H$_3$)$_2$), 24.1 (CH(**C**H$_3$)$_2$), 24.2 (CH(**C**H$_3$)$_2$), 24.2 (CH(**C**H$_3$)$_2$), 24.5 (CH(**C**H$_3$)$_2$), 24.6 (CH(**C**H$_3$)$_2$), 24.7-24.8 (overlapping (4x CH(**C**H$_3$)$_2$), 24.9 (CH(**C**H$_3$)$_2$), 26.0 (CH(**C**H$_3$)$_2$), 27.1 (CH(**C**H$_3$)$_2$), 27.9 (CH(**C**H$_3$)$_2$), 28.0 (CH(**C**H$_3$)$_2$), 28.2 (CH(**C**H$_3$)$_2$), 28.3 (**C**H(CH_3)$_2$), 28.4 (**C**H(CH_3)$_2$), 28.6 (**C**H(CH_3)$_2$), 28.7 (**C**H(CH_3)$_2$), 92.3 (**C**$_6$H$_6$), 95.9 ((**C**H)C(CH_3)$_2$), 96.1 ((**C**H)C(CH_3)$_2$), 123.5 (Ar-**C**), 124.0 (Ar-**C**), 124.1 (Ar-**C**), 124.5 (Ar-**C**), 125.4 (Ar-**C**), 125.5 (Ar-**C**), 125.9 (Ar-**C**), 142.9 (Ar-**C**), 143.6 (Ar-**C**), 145.7 (Ar-**C**), 146.2 (Ar-**C**), 146.7 (Ar-**C**), 169.1 (N**C**(CH_3)), 169.2 (N**C**(CH_3)), 169.4 (2x N**C**(CH_3)), 233.3 (Cr-**C**(H)O). *Quaternary ^{13}C resonance of the **C**$_{ipso}$ and **C**$_{ortho}$ atoms on the 2,6-diisopropylphenyl and Ir-**CO**-Mg unit not observed.*

IR (ATR, cm^{-1}), ν_{CO}: 1674 (s, CrCOMg), 1916 (s, CrCO); ν_{CH}: 2546 (w, CrCHO).

Anal. Calc. ($CrC_{67}H_{90}N_4O_3Mg_2$): C, 73.15; H, 8.25; N, 5.09. Found: C, 72.51; H, 8.39; N, 4.85.

Synthesis of **3e**

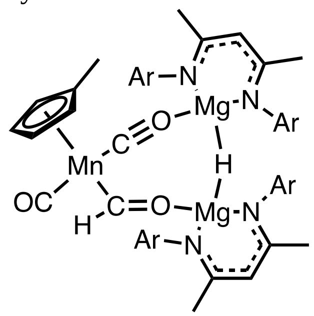

In a N_2 filled glovebox, a suspension of **1** (100 mg, 0.113 mmol) in toluene (3 mL) was added to a solution of [Mn(η^5-C_5H_4Me)(CO)$_3$] (17.9 uL, 24.7 mg, 0.113 mmol) in toluene (2 mL) and the resulting yellow solution stirred at 25 °C for 10 minutes. The solution was dried under vacuum, the crude product was extracted with *n*-hexane (3 x 1 mL) and filtered through a glass fibre. Recrystallisation from a toluene/*n*-pentane (1:1 v:v) mixture at -35 °C yielded yellow block crystals suitable for X-ray diffraction studies. The mother liquor was decanted, and the crystals washed with *n*-pentane (3 x 1 mL) and dried under vacuum to yield **3e** as yellow needles (84 mg, 0.077 mmol, 68 %).

^{1}H NMR (400 MHz, C_6D_6) δ: 0.17 (broad d, 3H, CH(C**H**$_3$)$_2$), 0.56 (broad d, 3H, CH(C**H**$_3$)$_2$), 0.85-0.91 (overlapping m, 12H, CH(C**H**$_3$)$_2$), 1.13-1.30 (overlapping m, 30H, CH(C**H**$_3$)$_2$), 1.20 (s, 3H, C**H**$_3$C$_5$H$_4$), 1.50-1.62 (overlapping d, 12H, NC(C**H**$_3$)), 2.87 (broad s, 1H, C**H**(CH$_3$)$_2$), 3.01 (broad s, 1H, C**H**(CH$_3$)$_2$), 3.17 (s, 1H, Mg-**H**-Mg), 3.24 (sept, $^3J_{H\text{-}H}$ = 6.7 Hz, 2H, C**H**(CH$_3$)$_2$), 3.45 (sept, $^3J_{H\text{-}H}$ = 6.5 Hz, 1H, C**H**(CH$_3$)$_2$), 3.56 (sept, $^3J_{H\text{-}H}$ = 6.5 Hz, 1H, C**H**(CH$_3$)$_2$), 3.76 (s, 2H, CH$_3$C$_5$**H**$_4$), 3.86 (broad s, 1H, C**H**(CH$_3$)$_2$), 3.91 (broad s, 1H, C**H**(CH$_3$)$_2$), 4.01 (s, 2H, CH$_3$C$_5$**H**$_4$), 4.81 (s, 1H, (CH$_3$)C(C**H**)C(CH$_3$)), 4.86 (s, 1H, (CH$_3$)C(C**H**)C(CH$_3$)), 6.96-7.22 (12H, Ar-**H**), 14.53 (s, 1H, Mn-C**H**O).

^{13}C NMR (101 MHz, C_6D_6) δ: δ 12.7 (5x **C**H$_3$ in Cp'), 22.7 (2x NC(**C**H$_3$)), 23.0 (2x NC(**C**H$_3$)), 23.9 (CH(**C**H$_3$)$_2$), 24.1 (CH(**C**H$_3$)$_2$), 24.2 (CH(**C**H$_3$)$_2$), 24.3 (CH(**C**H$_3$)$_2$), 24.7 (6x CH(**C**H$_3$)$_2$), 27.0 (CH(**C**H$_3$)$_2$), 27.1 (2x CH(**C**H$_3$)$_2$), 27.8 (CH(**C**H$_3$)$_2$), 28.0 (CH(**C**H$_3$)$_2$), 28.2 (CH(**C**H$_3$)$_2$), 28.7 (2x **C**H(CH$_3$)$_2$), 28.5 (2x **C**H(CH$_3$)$_2$), 85.1 (CH$_3$**C**$_5$H$_4$), 86.8 (CH$_3$**C**$_5$H$_4$), 87.7 (CH$_3$**C**$_5$H$_4$), 96.0 ((**C**H)C(CH$_3$)$_2$), 96.2 ((**C**H)C(CH$_3$)$_2$), 123.2 (Ar-**C**), 123.7 (Ar-**C**), 123.9 (Ar-**C**), 124.0 (Ar-**C**), 124.2 (Ar-**C**), 124.4 (Ar-**C**), 124.7 (Ar-**C**), 125.7 (Ar-**C**), 125.9 (Ar-**C**), 126.2 (Ar-**C**), 142.3 (Ar-**C**), 142.6 (Ar-**C**), 142.7 (Ar-**C**), 142.8 (Ar-**C**), 143.1 (Ar-**C**), 143.5 (Ar-**C**), 143.3 (Ar-**C**), 144.9 (Ar-**C**), 145.8 (Ar-**C**), 146.0 (Ar-**C**), 168.4 (N**C**(CH$_3$)), 169.5 (N**C**(CH$_3$)), 169.7 (N**C**(CH$_3$)), 169.8 (N**C**(CH$_3$)), 226.4 (Mn-**C**O-Mg), 327.7 (Mn-**C**HO). Q*uaternary ^{13}C resonance of the* ***C**$_{ipso}$* *atoms on the 2,6-diisopropylphenyl unit not observed.*

IR (ATR, cm^{-1}), ν_{CO}: 1733 (m, MnCOMg), 1949 (m, MnCO).

Anal. Calc. ($MnC_{67}H_{91}N_4O_3Mg_2$), 72.89; H, 8.31; N, 5.08. Found: C, 72.26; H, 8.48; N, 5.16.

Synthesis of **3f**

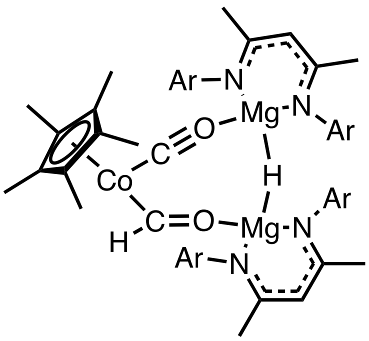

In a N_2 filled glovebox, a suspension of **1** (100 mg, 0.113 mmol) in toluene (3 mL) was added to a solution of [Co(η^5-Cp*)(CO)$_2$] (28.3 mg, 0.113 mmol) in toluene (2 mL) and the resulting green-black solution stirred at 25 °C for 30 minutes. The solution was dried under vacuum, the crude product was extracted with *n*-hexane (3 x 1 mL) and filtered through a glass fibre. Recrystallisation from a toluene solution at -35 °C yielded orange block crystals suitable for x-ray diffraction studies. The mother liquor was decanted, and the crystals washed with *n*-pentane (3 x 1 mL) and dried under vacuum to yield **3f** as orange blocks (27 mg, 0.024 mmol, 21 %).

^{1}H NMR (400 MHz, C_6D_6) δ: 0.48 (d, $^3J_{H-H}$ = 6.8 Hz, 3H, CH(C**H**$_3$)$_2$), 0.67 (d, $^3J_{H-H}$ = 6.8 Hz, 3H, CH(C**H**$_3$)$_2$), 0.89 (m, $^3J_{H-H}$ = 6.7 Hz, 3H, CH(C**H**$_3$)$_2$), 0.98 (m, $^3J_{H-H}$ = 6.7 Hz, 3H, CH(C**H**$_3$)$_2$), 1.16 (d, $^3J_{H-H}$ = 6.8 Hz, 3H, CH(C**H**$_3$)$_2$), 1.20-1.40 (overlapping m, 33H, CH(C**H**$_3$)$_2$), 1.51-1.55 (overlapping s, 12H, NC(C**H**$_3$)), 1.59 (s, 15H, Cp*), 2.67 (sept, $^3J_{H-H}$ = 6.8 Hz, 1H, C**H**(CH$_3$)$_2$), 2.76 (sept, $^3J_{H-H}$ = 6.8 Hz, 1H, C**H**(CH$_3$)$_2$), 2.99 (s, 1H, Mg-**H**-Mg), 3.10-3.28 (m, 4H, C**H**(CH$_3$)$_2$), 3.47 (m, 2H, C**H**(CH$_3$)$_2$), 4.82 (s, 1H, (CH$_3$)C(C**H**)C(CH$_3$), 4.84 (s, 1H, (CH$_3$)C(C**H**)C(CH$_3$)), 6.98-7.21 (12H, Ar-**H**), 13.20 (s, 1H, Co-C**H**O).

^{13}C NMR (101 MHz, C_6D_6) δ: 10.4 (5x CH$_3$ in Cp*), 23.3 (2x NC(**C**H$_3$)), 23.6 (2x NC(**C**H$_3$)), 23.9 (CH(**C**H$_3$)$_2$), 24.0 (CH(**C**H$_3$)$_2$), 24.3 (CH(**C**H$_3$)$_2$), 24.4 (CH(**C**H$_3$)$_2$), 24.5 (CH(**C**H$_3$)$_2$), 24.6 (CH(**C**H$_3$)$_2$), 24.7 (CH(**C**H$_3$)$_2$), 24.9 (CH(**C**H$_3$)$_2$), 25.2 (CH(**C**H$_3$)$_2$), 26.0 (CH(**C**H$_3$)$_2$), 27.0 (CH(**C**H$_3$)$_2$), 27.7 (CH(**C**H$_3$)$_2$), 27.9 (CH(**C**H$_3$)$_2$), 28.0 (CH(**C**H$_3$)$_2$), 28.1 (CH(**C**H$_3$)$_2$), 28.2 (**C**H(CH$_3$)$_2$), 28.3 (**C**H(CH$_3$)$_2$), 28.7 (2x **C**H(CH$_3$)$_2$), 29.0 (**C**H(CH$_3$)$_2$), 95.7 ((**C**H)C(CH$_3$)$_2$), 95.8 (5x CCH$_3$ in Cp*), 96.1 ((**C**H)C(CH$_3$)$_2$), 123.0 (Ar-**C**), 123.2 (Ar-**C**), 123.6 (Ar-**C**), 123.7 (Ar-**C**), 124.1 (Ar-**C**), 124.2 (Ar-C), 124.6 (Ar-**C**), 124.8 (Ar-**C**), 125.0 (Ar-**C**), 125.6 (2x Ar-**C**), 125.7 (Ar-**C**), 126.0 (Ar-**C**), 142.3 (Ar-**C**), 142.5 (Ar-**C**), 142.6 (Ar-**C**), 142.9 (Ar-**C**), 143.1 (Ar-**C**), 143.2 (Ar-**C**), 143.5 (Ar-**C**), 144.7 (Ar-**C**), 144.8 (Ar-**C**), 145.7 (Ar-**C**), 145.8 (Ar-**C**), 168.3 (N**C**(CH$_3$)), 169.1 (2x (N**C**(CH$_3$)), 169.9 (N**C**(CH$_3$)), 300.5 (Co-**C**HO).

IR (ATR, cm^{-1}), ν_{CO}: 1791 (s, CoCOMg); ν_{CH}: 2583 (w, CoCHO).

Anal. Calc. ($CoC_{70}H_{99}N_4O_2Mg_2$): C, 74.00; H, 8.78; N, 4.93. Found: C, 74.52; H, 9.29; N, 4.84.

Synthesis of **3g**

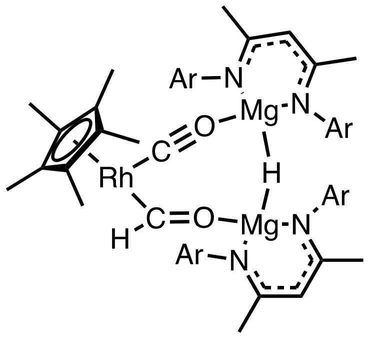

In a N_2 filled glovebox, a suspension of **1** (50 mg, 0.057 mmol) in toluene (2 mL) was added to a solution of [Rh(η^5-Cp*)(CO)$_2$] (16.6 mg, 0.057 mmol) in toluene (2 mL) and the resulting green-black solution stirred at 25 °C for 30 minutes. The solution was dried under vacuum, the crude product was extracted with *n*-hexane (3 x 1 mL) and filtered through a glass fibre. Recrystallisation from a toluene/*n*-hexane (1:1 v:v) mixture at -35 °C yielded orange block crystals suitable for x-ray diffraction studies. The mother liquor was decanted, and the crystals washed with *n*-pentane (3 x 1 mL) and dried under vacuum to yield **3g** as orange blocks (45 mg, 0.038 mmol, 67 %).

^{1}H NMR (400 MHz, C_6D_6) δ: 0.37 (d, $^3J_{H-H}$ = 6.8 Hz, 3H, CH(C**H**$_3$)$_2$), 0.72 (d, $^3J_{H-H}$ = 6.8 Hz, 3H, CH(C**H**$_3$)$_2$), 0.89 (multiplet, 6H, CH(C**H**$_3$)$_2$), 0.96 (d, $^3J_{H-H}$ = 6.6 Hz, 3H, CH(C**H**$_3$)$_2$), 1.02 (d, $^3J_{H-H}$ = 6.7 Hz, 3H, CH(C**H**$_3$)$_2$), 1.15 (d, $^3J_{H-H}$ = 6.8 Hz 3H, CH(C**H**$_3$)$_2$), 1.19-1.41 (overlapping multiplets, 27H, CH(C**H**$_3$)$_2$), 1.53-1.60 (overlapping singlets, 9H, NC(C**H**$_3$)), 1.64 (s, 3H, NC(C**H**$_3$)), 1.78 (s, 15H, Cp*), 2.72 (sept, $^3J_{H-H}$ = 6.7 Hz, 2H, C**H**(CH$_3$)$_2$), 2.99 (s, 1H, Mg-**H**-Mg), 3.19 (sept, $^3J_{H-H}$ = 7.0 Hz, 3H, C**H**(CH$_3$)$_2$), 3.45 (sept, $^3J_{H-H}$ = 6.5 Hz, 2H, C**H**(CH$_3$)$_2$), 3.55 (sept, $^3J_{H-H}$ = 6.7 Hz, 1H, C**H**(CH$_3$)$_2$), 4.80 (s, 1H, (CH$_3$)C(C**H**)C(CH$_3$)), 4.84 (s, 1H, (CH$_3$)C(C**H**)C(CH$_3$)), 6.92-7.20 (12H, Ar-**H**), 13.05 (d, $^2J_{Rh-H}$ = 2.1 Hz, 1H, Rh-C**H**O).

^{13}C NMR (101 MHz, C_6D_6) δ: 10.7 (5x CH$_3$ in Cp*), 23.1 (NC(**C**H$_3$)), 23.2 (NC(**C**H$_3$)), 23.8 (NC(**C**H$_3$)), 23.9 (NC(**C**H$_3$)), 24.1 (CH(**C**H$_3$)$_2$), 24.4 (CH(**C**H$_3$)$_2$), 24.5 (CH(**C**H$_3$)$_2$), 24.6 (CH(CH3)2), 24.7 (CH(**C**H$_3$)$_2$), 24.8 (CH(**C**H$_3$)$_2$), 24.9 (CH(CH3)2), 25.1 (CH(**C**H$_3$)$_2$), 26.8 (CH(**C**H$_3$)$_2$), 26.9 (CH(**C**H$_3$)$_2$), 27.7 (CH(**C**H$_3$)$_2$), 27.9 (CH(**C**H$_3$)$_2$), 28.1 (CH(**C**H$_3$)$_2$), 28.3 (CH(**C**H$_3$)$_2$), 28.4 (CH(**C**H$_3$)$_2$), 28.5 (CH(**C**H$_3$)$_2$), 28.6 (**C**H(CH$_3$)$_2$), 28.7 (**C**H(CH$_3$)$_2$), 28.8 (**C**H(CH$_3$)$_2$), 29.0 (**C**H(CH$_3$)$_2$), 95.6 ((**C**H)C(CH$_3$)$_2$), 95.9 ((**C**H)C(CH$_3$)$_2$), 99.9 (5x CCH$_3$ in Cp*), 123.0 (Ar-**C**), 123.2 (Ar-**C**), 123.5 (Ar-**C**), 123.8 (Ar-**C**), 124.2 (Ar-**C**), 124.3 (Ar-**C**), 124.5 (Ar-**C**), 124.8 (Ar-**C**), 125.1 (Ar-**C**), 125.6 (2x Ar-**C**), 125.9 (Ar-**C**), 142.3 (2x Ar-**C**), 142.5 (Ar-**C**), 142.9 (Ar-**C**),143.0 (Ar-**C**), 143.2 (Ar-**C**), 143.4 (2x Ar-**C**), 144.4 (Ar-**C**), 144.7 (Ar-**C**), 145.5 (Ar-**C**), 145.6 (Ar-**C**), 168.3 (N**C**(CH$_3$)), 169.1 (N**C**(CH$_3$)), 169.2 ((N**C**(CH$_3$)), 170.0 (N**C**(CH$_3$)), 285.3 (s, $^1J_{Rh-C}$ = 56.0 Hz, Rh-**C**HO).

IR (ATR, cm^{-1}), ν_{CO}: 1784 (s, RhCOMg); ν_{CH}: 2585 (w, RhCHO).

Anal. Calc. ($RhC_{70}H_{99}N_4O_2Mg_2$): C, 71.24; H, 8.46; N, 4.75. Found: C, 71.24; H, 8.51; N, 4.77.

Synthesis of **3h**

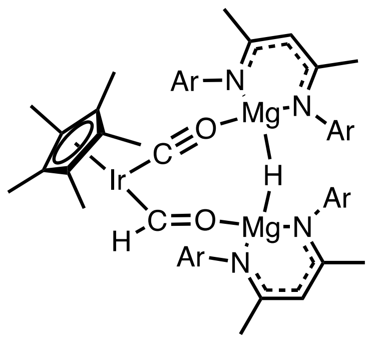

In a N_2 filled glovebox, a suspension of **1** (50 mg, 0.057 mmol) in toluene (2 mL) was added to a solution of [Ir(η^5-Cp*)(CO)$_2$] (21.7 mg, 0.057 mmol) in toluene (2 mL) and the resulting yellow solution stirred at 25 °C for 30 minutes. The solution was dried under vacuum, the crude product was extracted with *n*-hexane (3 x 1 mL) and filtered through a glass fibre. Recrystallisation from toluene solution at -35 °C yielded yellow block crystals suitable for x-ray diffraction studies. The mother liquor was decanted, and the crystals washed with *n*-pentane (3 x 1 mL) and dried under vacuum to yield **3h** as yellow blocks (35 mg, 0.028 mmol, 49 %).

^{1}H NMR (400 MHz, C_6D_6) δ: 0.44 (d, $^3J_{H-H}$ = 6.7 Hz, 3H, CH(C**H**$_3$)$_2$), 0.77 (d, $^3J_{H-H}$ = 6.7 Hz, 3H, CH(C**H**$_3$)$_2$), 0.89 (m, 6H, CH(C**H**$_3$)$_2$), 0.97 (d, $^3J_{H-H}$ = 6.6 Hz, 3H, CH(C**H**$_3$)$_2$), 1.02 (d, $^3J_{H-H}$ = 6.6 Hz, 3H, CH(C**H**$_3$)$_2$), 1.17-1.42 (overlapping m, 30H, CH(C**H**$_3$)$_2$), 1.55-1.61 (overlapping s, 12H, NC(C**H**$_3$)), 1.75 (s, 15H, Cp*), 2.75 (sept, $^3J_{H-H}$ = 6.6 Hz, 2H, C**H**(CH$_3$)$_2$), 3.13 (s, 1H, Mg-**H**-Mg), 3.26 (sept, $^3J_{H-H}$ = 6.8 Hz, 3H, C**H**(CH$_3$)$_2$), 3.47 (sept, $^3J_{H-H}$ = 7.4 Hz, 2H, C**H**(CH$_3$)$_2$), 3.57 (sept, $^3J_{H-H}$ = 7.2 Hz, 1H, C**H**(CH$_3$)$_2$), 4.82 (s, 1H, (CH$_3$)C(C**H**)C(CH$_3$)), 4.84 (s, 1H, (CH$_3$)C(C**H**)C(CH$_3$)), 7.00-7.21 (12H, Ar-**H**), 13.53 (s, 1H, Ir-C**H**O).

^{13}C NMR (101 MHz, C_6D_6) δ: 10.4 (5x CH$_3$ in Cp*), 22.7 (NC(**C**H$_3$)), 23.6 (NC(**C**H$_3$)), 23.8 (NC(**C**H$_3$)), 23.9 (CH(**C**H$_3$)$_2$), 24.0 (CH(**C**H$_3$)$_2$), 24.1 (CH(**C**H$_3$)$_2$), 24.4 (CH(**C**H$_3$)$_2$), 24.5 (CH(**C**H$_3$)$_2$), 24.7 (CH(**C**H$_3$)$_2$), 24.8 (CH(**C**H$_3$)$_2$), 24.9 (CH(**C**H$_3$)$_2$), 25.1 (CH(**C**H$_3$)$_2$), 25.2 (CH(**C**H$_3$)$_2$), 26.9 (CH(**C**H$_3$)$_2$), 27.0 (CH(CH3)2), 27.7 (CH(**C**H$_3$)$_2$), 27.9 (CH(**C**H$_3$)$_2$), 28.1 (CH(**C**H$_3$)$_2$, 28.4 (CH(**C**H$_3$)$_2$), 28.6 (**C**H(CH$_3$)$_2$), 28.7 (**C**H(CH$_3$)$_2$), 28.8 (**C**H(CH$_3$)$_2$), 29.0 (**C**H(CH$_3$)$_2$), 95.5 ((**C**H)C(CH$_3$)$_2$), 95.8 ((**C**H)C(CH$_3$)$_2$), 97.9 (5x CCH$_3$ in Cp*), 122.9 (Ar-**C**), 123.2 (Ar-**C**), 123.6 (Ar-**C**), 123.9 (Ar-**C**), 124.1 (Ar-**C**), 124.2 (Ar-**C**), 124.5 (Ar-**C**), 124.8 (2x Ar-**C**), 125.3 (Ar-**C**), 125.5 (Ar-**C**), 125.8 (Ar-**C**), 142.4 (2x Ar-**C**), 142.5 (Ar-**C**), 142.9 (Ar-**C**),143.0 (Ar-**C**), 143.3 (Ar-**C**), 143.5 (2x Ar-**C**), 144.8 (Ar-**C**), 144.9 (Ar-**C**), 145.8 (Ar-**C**), 145.9 (Ar-**C**), 168.1 (N**C**(CH$_3$)), 168.9 (N**C**(CH$_3$)), 169.0 ((N**C**(CH$_3$)), 169.8 (N**C**(CH$_3$)), 244.5 (s, Ir-**C**HO). *Quaternary ^{13}C resonance of the Ir-**C**O-Mg group not observed.*

IR (ATR, cm^{-1}), ν_{CO}: 1785 (s, IrCOMg); ν_{CH}: 2635 (w, IrCHO).

Anal. Calc. ($IrC_{70}H_{99}N_4O_2Mg_2$): C, 66.23; H, 7.86; N, 4.41. Found: C, 66.28; H, 8.20; N, 4.74.

Synthesis of **4a**

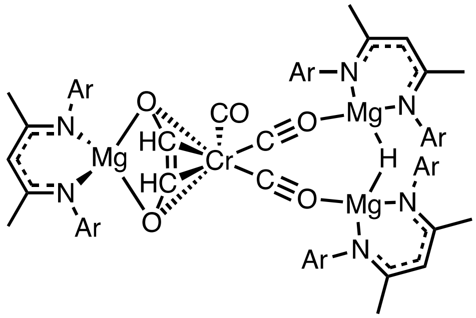

Route **A**: In a N_2 filled glovebox, a solution of **3a** (10 mg, 0.009 mmol) in C_6D_6 (0.6 mL) was transfered to a J-Youngs NMR tube and the resulting yellow solution held at 25 °C overnight. The solution was dried under vacuum, the crude product was extracted with *n*-hexane (3 x 1 mL) and filtered through a glass fibre. Recrystallisation from *n*-hexane solution at -35 °C yielded yellow block crystals suitable for x-ray diffraction studies. The mother liquor was decanted, and the crystals washed with *n*-pentane (3 x 0.5 mL) and dried under vacuum to yield **4a** as yellow blocks (62 % NMR yield; 4.2 mg, 0.003 mmol, 31 % isolated yield).

Route **B**: In a N_2 filled glovebox, a solution of **3a** (20 mg, 0.018 mmol) in C_6D_6 (0.4 mL) was transfered to a J-Youngs NMR tube. A solution of **1** (8 mg, 0.009 mmol) in C_6D_6 (0.4 mL) was added and the resulting yellow solution held at 25 °C overnight. The solution was dried under vacuum, the crude product was extracted with *n*-hexane (3 x 1 mL) and filtered through a glass fibre. Recrystallisation from *n*-hexane solution at -35 °C yielded yellow block crystals. The mother liquor was decanted, and the crystals washed with *n*-pentane (3 x 0.5 mL) and dried under vacuum to yield **4a** as yellow blocks (70 % NMR yield; 10.4 mg, 0.007 mmol, 38 % isolated yield).

Route **C**: In a N_2 filled glovebox, a suspension of **1** (100 mg, 0.113 mmol) in toluene (2 mL) was added to a solution of [$Cr(CO)_6$] (16.6 mg, 0.0754 mmol) in toluene (2 mL) and the resulting yellow-brown solution stirred at 25 °C overnight. The solution was dried under vacuum, the crude product was extracted with *n*-hexane (3 x 1 mL) and filtered through a glass fibre. Recrystallisation from *n*-hexane solution at -35 °C yielded yellow block crystals suitable for x-ray diffraction studies. The mother liquor was decanted, and the crystals washed with *n*-pentane (3 x 1 mL) and dried under vacuum to yield **4a** as yellow-brown blocks (58 mg, 0.04 mmol, 51 %).

^{1}H NMR (400 MHz, C_6D_6) δ: 0.20 (d, $^3J_{H-H}$ = 6.7 Hz, 3H, CH(C**H**$_3$)$_2$), 0.27 (d, $^3J_{H-H}$ = 6.8 Hz, 3H, CH(C**H**$_3$)$_2$), 0.86-0.91 (overlapping doublets, 12H, CH(C**H**$_3$)$_2$), 1.04 (d, $^3J_{H-H}$ = 6.7 Hz, 3H, CH(C**H**$_3$)$_2$), 1.08-1.14 (overlapping doublets, 12H, CH(C**H**$_3$)$_2$), 1.20-1.29 (overlapping doublets, 15H, CH(C**H**$_3$)$_2$), 1.34 (d, $^3J_{H-H}$ = 6.7 Hz, 6H, CH(C**H**$_3$)$_2$), 1.38 (d, $^3J_{H-H}$ = 6.7 Hz, 6H, CH(C**H**$_3$)$_2$), 1.42-1.44 (overlapping doublets, 12H, CH(C**H**$_3$)$_2$), 1.46 (s, 9H, NC(C**H**$_3$)), 1.54 (s, 3H, NC(C**H**$_3$)), 1.63 (s, 6H, NC(C**H**$_3$)), 2.72 (s, 1H, Mg-**H**-Mg), 2.78 (sept, $^3J_{H-H}$ = 6.8 Hz, 2H, C**H**(CH$_3$)$_2$), 3.12 (sept, $^3J_{H-H}$ = 6.8 Hz, 7H, C**H**(CH$_3$)$_2$), 3.28 ((sept,

$^{3}J_{H\text{-}H}$ = 6.8 Hz, 3H, C**H**(CH_3)$_2$), 4.33 (s, 1H, **H**(O)CC(O)H), 4.73 (s, 1H, (CH_3)C(C**H**)C(CH_3))), 4.74 (s, 1H, (CH_3)C(C**H**)C(CH_3))), 4.84 (s, 1H, (CH_3)C(C**H**)C(CH_3))), 5.45 (s, 1H, **H**(O)CC(O)H), 6.97-7.37 (18H, Ar-**H**).

^{13}C NMR (101 MHz, C_6D_6) δ: 22.5 (NC(**C**H_3)), 22.7 (2x NC(**C**H_3)), 22.9 (NC(**C**H_3)), 23.0 (2x NC(**C**H_3)), 23.8 (4x CH(**C**H_3)$_2$), 23.9 (CH(**C**H_3)$_2$), 24.0 (CH(**C**H_3)$_2$), 24.5 (6x CH(**C**H_3)$_2$), 24.6 (4x CH(**C**H_3)$_2$), 24.7 (2x CH(**C**H_3)$_2$), 24.8 (CH(**C**H_3)$_2$), 25.0 (2x CH(**C**H_3)$_2$), 25.2 (CH(**C**H_3)$_2$), 26.6 (CH(**C**H_3)$_2$), 26.7 (CH(**C**H_3)$_2$), 27.8 (2x (**C**H(CH_3)$_2$), 28.1 (2x (**C**H(CH_3)$_2$), 28.2 (2x (**C**H(CH_3)$_2$), 28.4 (2x (**C**H(CH_3)$_2$), 28.5 (2x (**C**H(CH_3)$_2$), 28.7 (2x (**C**H(CH_3)$_2$), 94.3 ((**C**H)C(CH_3)$_2$), 95.8 ((**C**H)C(CH_3)$_2$), 96.0 ((**C**H)C(CH_3)$_2$), 110.2 (H(O)**C**C(O)H)), 110.5 (H(O)**C**C(O)H), 122.7 (Ar-**C**), 124.1 (Ar-**C**), 124.2 (Ar-**C**), 124.3 (Ar-**C**), 124.4 (Ar-**C**), 124.5 (Ar-**C**), 125.8 (Ar-**C**), 126.0 (Ar-**C**), 126.1 (Ar-**C**), 126.3 (Ar-**C**), 126.4 (Ar-**C**), 142.0 (Ar-**C**), 142.2 (Ar-**C**), 142.4 (Ar-**C**), 142.5 (Ar-**C**), 142.9 (Ar-**C**), 143.0 (Ar-**C**), 143.2 (Ar-**C**), 143.7 (Ar-**C**), 144.0 (Ar-**C**), 144.5 (Ar-**C**), 144.6 (Ar-**C**), 145.0 (Ar-**C**), 145.2 (Ar-**C**), 168.9 (N**C**(CH_3)), 169.3 (2x N**C**(CH_3)), 169.4 (N**C**(CH_3)), 169.9 (N**C**(CH_3)), 170.2 (N**C**(CH_3)), 219.60 (Cr-**C**O), 242.9 (Cr-**C**OMg), 243.8 (Cr-**C**OMg). *Twelve quaternary ^{13}C resonance of the Ar-**C**$_{ortho}$ groups not observed.*

IR (ATR, cm^{-1}), ν_{CO}: 1647 (s, CrCOMg), 1744 (s, CrCOMg), 1982 (s, CrCO).

3. X-ray Data

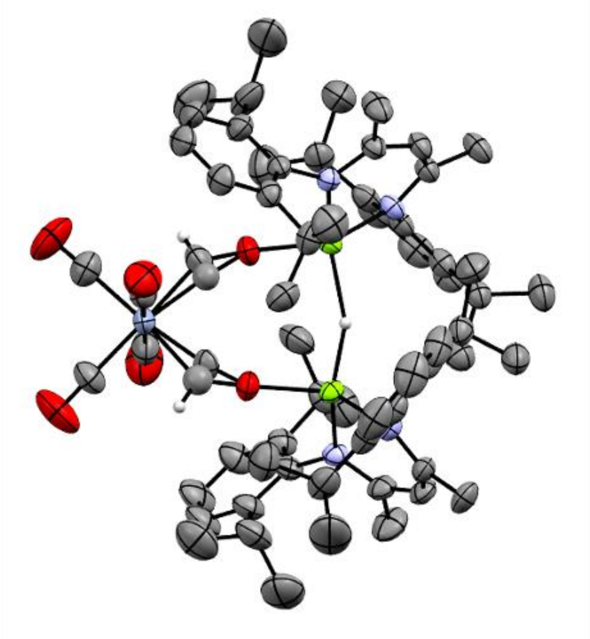

Figure S1. The X-ray crystal structure of **3a**. Most hydrogen atoms and included toluene molecule omitted for clarity.

3a was found to crystallise in the space group Pna2_1. The C61-based C(H)–O unit and the C62-based C=O moiety in the structure of **3a** were found to be disordered. Two orientations were identified of *ca*. 62 and 38% occupancy, with the minor occupancy orientation representing a "flipping" of the C(H)–O and C=O groups. (As the structure was presumed to always be a "mixed" C(H)–O/C=O species, the disorders of the two groups were treated as linked.) The thermal parameters of adjacent atoms were restrained to be similar, and only the non-hydrogen atoms of the major occupancy orientation were refined anisotropically (those of the minor occupancy orientation were refined isotropically). The included toluene solvent molecule was found to be disordered. Two orientations were identified of *ca*. 53 and 47% occupancy, their geometries were optimised, the thermal parameters of adjacent atoms were restrained to be similar, and only the non-hydrogen atoms of the major occupancy orientation were refined anisotropically (those of the minor occupancy orientation were refined isotropically). The Mg–H–Mg bridging hydrogen atom was located from a ΔF map and refined freely. The absolute structure of **3a** was determined by use of the Flack parameter [x = –0.002(4)].

Crystal Data for $C_{71}H_{92}CrMg_2N_4O_6$, M =1198.10, orthorhombic, space group Pna2_1 (no. 33), a = 35.2188(4) Å, b = 15.39140(16) Å, c = 12.77462(11) Å, V = 6924.70(12) Å^3, Z = 4, ρ_{calc}g/cm^3 = 1.149,

μ(CuKα) = 1.934 mm^{-1}, T = 173(2), Yellow plates, F^2refinement, R_1(obs) = 0.0376, wR_2(all) = 0.0978, 9749 independent observed reflections (R_{int} = 0.0276), 8954 independent measured reflections [$|F_o| > 4\sigma(|F_o|)$, $2\theta_{full}$ = 147.518], 797 parameters. CCDC no. 2116002.

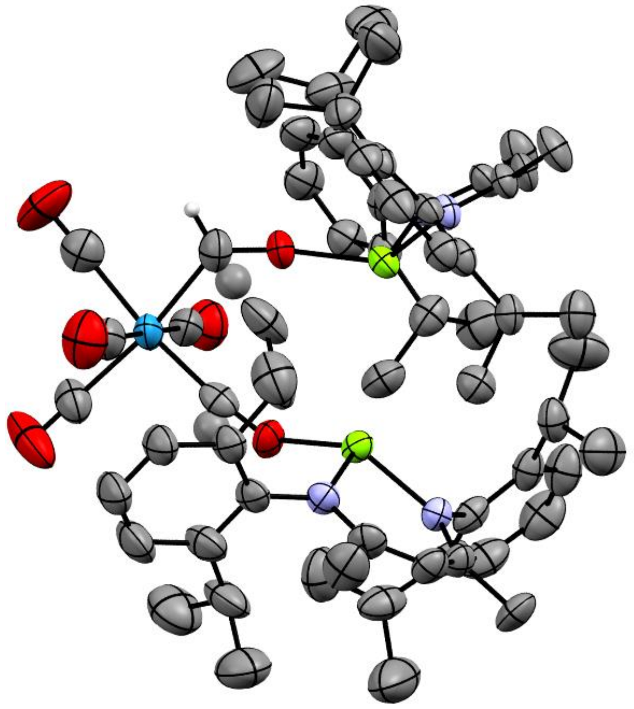

Figure S2. The X-ray crystal structure of **3b**. Most hydrogen atoms and included toluene molecules omitted for clarity.

3c was found to crystallise in the space group $Pna2_1$. with two included toluene molecules in the asymmetric unit. The C61-based C(H)–O unit and the C62-based C=O moiety in the structure of **3c** were found to be disordered. Two orientations were identified of *ca*. 55 and 45% occupancy, with the minor occupancy orientation representing a "flipping" of the C(H)–O and C=O groups. (As the structure was presumed to always be a "mixed" C(H)–O/C=O species, the disorders of the two groups were treated as linked.) The thermal parameters of adjacent atoms were restrained to be similar, and only the non-hydrogen atoms of the major occupancy orientation were refined anisotropically (those of the minor occupancy orientation were refined isotropically). The included toluene solvent molecule was found to be disordered. Three orientations were identified of *ca*. 51, 36 and 13% occupancy, their geometries were optimised, the thermal parameters of adjacent atoms were restrained to be similar, and only the non-hydrogen atoms of the major occupancy orientation were refined anisotropically (those of the minor occupancy orientations were refined isotropically). The Mg–H–Mg bridging hydrogen atom could not be reliably located and so this atom was omitted, making the atom list for the asymmetric unit low by 1H (and that for the unit cell low by 4H). (A significant electron density peak was found at the expected location from a ΔF map, but when refined freely as a hydrogen atom its isotropic thermal parameter would go negative. Further, when assigned a sensible

fixed thermal parameter the hydrogen atom would move away from the “correct” site and a new residual electron density peak would appear back in the expected location.) The absolute structure of **3b** was determined by use of the Flack parameter [x = –0.042(12)].

Crystal Data for $C_{71}H_{92}WMg_2N_4O_6$, M =1329.95, orthorhombic, space group Pna2_1 (no. 33), a = 35.3464(8) Å, b = 15.4039(3) Å, c = 12.8690(3) Å, V = 7006.8(3) Å^3, Z = 4, ρ_{calc}g/cm^3 = 1.261, μ(CuKα) = 3.624 mm^{-1}, T = 173(2), Yellow blockss, F^2refinement, R_1(obs) = 0.0427, wR_2(all) = 0.1166, 9229 independent observed reflections (R_{int} = 0.0341), 7525 independent measured reflections [$|F_o| > 4\sigma(|F_o|)$, $2\theta_{full}$ = 147.05], 809 parameters. CCDC no. 2116003.

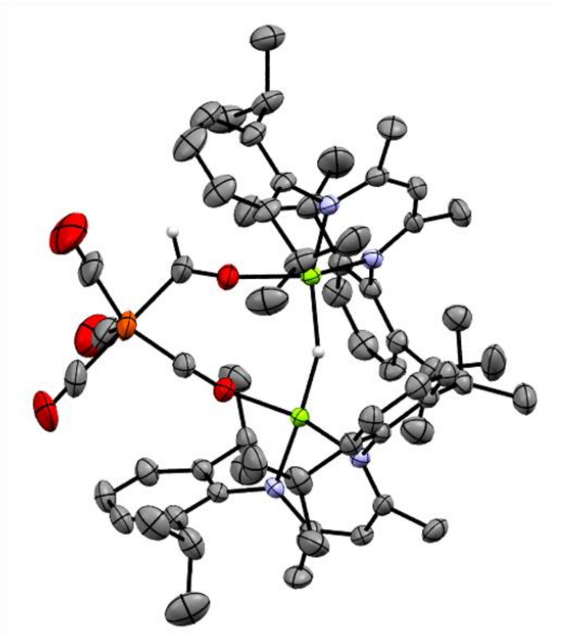

Figure S3. The X-ray crystal structure of **3c**. Most hydrogen atoms and included toluene molecules omitted for clarity.

3c was found to crystallise in the space group P-1., with two included toluene molecules in the asymmetric unit.

Crystal Data for $C_{63}H_{84}N_4Mg_2O_5Fe$, *M* =721.18, triclinic, space group P-1 (no. 2), *a* = 13.3957(10) Å, *b* = 13.4353(8) Å, *c* = 22.6744(8) Å, *α* = 99.136(4)°, *β* = 97.078(4)°, *γ* = 112.627(6)°, *V* = 3642.1(4) Å^3, *Z* = 3, ρ_{calc}g/cm^3= 0.986, μ(MoKα) = 0.265 mm^{-1}, *T* = 173.00(14), clear green blocks, F^2 refinement, R_1(obs) = 0.1081, wR_2(all) = 0.3347, 14369 independent observed reflections (R_{int} = 0.0288), 10076 independent measured reflections [$|F_o| > 4\sigma(|F_o|)$, $2\theta_{full}$ = 56.442], 752 parameters. CCDC no. 2116004.

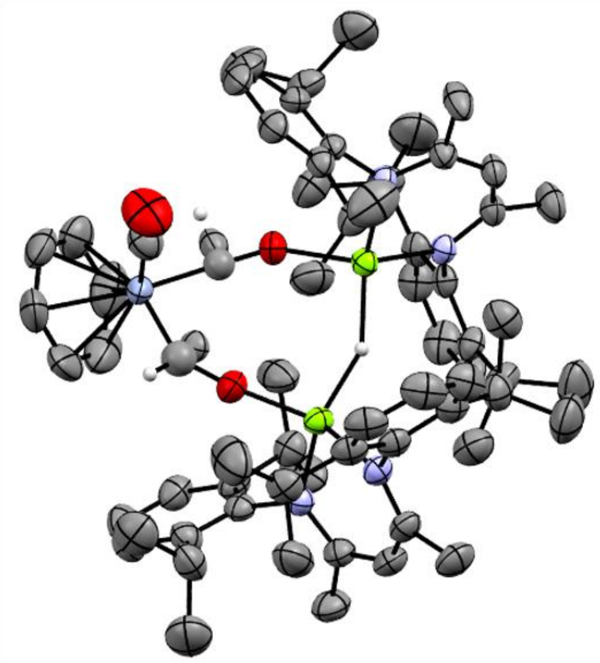

Figure S4. The X-ray crystal structure of **3d**. Most hydrogen atoms omitted for clarity.

3d was found to crystallise in the space group $P2_1/c$. The transition metal formyl carbon (C1, C2) was modelled as disordered over two sites in ca. 55:45 occupancies for the major and minor orientations respectively.

Crystal Data for $C_{67}H_{90}CrMg_2N_4O_3$, *M* =1100.04, monoclinic, space group $P2_1/c$ (no. 14), a = 15.3628(2) Å, b = 12.9497(2) Å, c = 35.8850(6) Å, β = 99.436(2)°, V = 7042.50(19) Å^3, Z = 4, ρ_{calc}g/cm^3 = 1.038, μ(Cu Kα) = 1.829 mm^{-1}, T = 173.00(14), Orange blocks, F^2 refinement, R_1(obs) = 0.0517, wR_2(all) = 0.1558, 13503 independent observed reflections (R_{int} = 0.0362), 9836 independent measured reflections [$|F_o| > 4\sigma(|F_o|)$, $2\theta_{full}$ = 146.75], 727 parameters. CCDC no. 2116005.

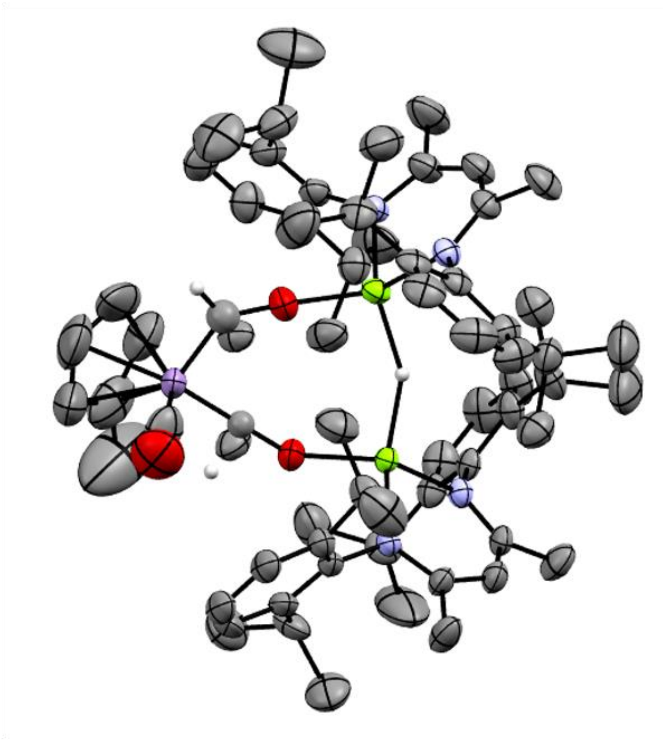

Figure S5. The X-ray crystal structure of **3e**. Most hydrogen atoms and included pentane molecule omitted for clarity.

3d was found to crystallise in the space group $P2_1/c$. with an included pentane molecule in the asymmetric unit. The transition metal formyl carbon (C1, C2) was modelled as disorder over two sites in ca. 52:48 occupancies for the major and minor orientations respectively.

Crystal Data for $C_{63}H_{84}N_4O_5Mg_2Mn$, M =1080.90, monoclinic, space group $P2_1/c$ (no. 14), a = 15.2588(7) Å, b = 12.9668(5) Å, c = 35.832(3) Å, β = 97.903(5)°, V = 7022.3(7) Å^3, Z = 5, ρ_{calc}g/cm^3 = 1.278, μ(Mo Kα) = 0.311 mm^{-1}, T = 172.95(10), yellow blocks, F^2refinement, R_1(obs) = 0.0923, wR_2(all) = 0.2921, 14105 independent observed reflections (R_{int} = 0.0410), 8681 independent measured reflections [$|F_o| > 4\sigma(|F_o|)$, $2\theta_{full}$ = 56.858], 750 parameters. CCDC no. 2116006.

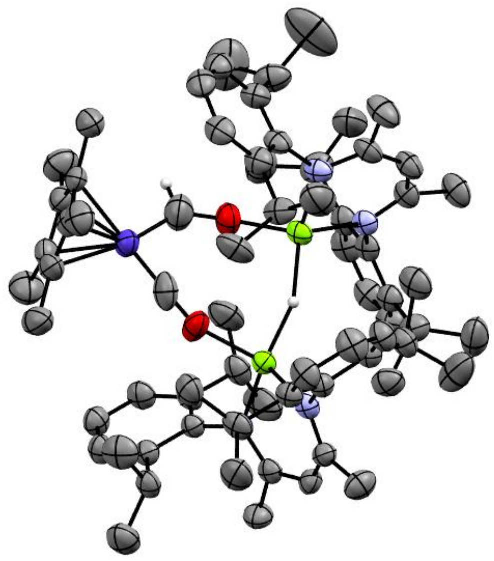

Figure S6. The X-ray crystal structure of **3f**. Most hydrogen atoms and included toluene molecules omitted for clarity.

3f was found to crystallise in the space group $P2_{1/c}$ space group, with two included toluene molecules in the aystmmetric unit. The C81- and C91-based included toluene solvent molecules in the structure of **3f** were found to be disordered, and in each case three orientations were identified, of *ca*. 53:26:21 and 55:33:12% occupancy respectively. The geometries of each set of orientations were optimised, the thermal parameters of adjacent atoms were restrained to be similar, and only the non-hydrogen atoms of the two major occupancy orientations were refined anisotropically (those of the minor occupancy orientations were refined isotropically). The Mg–H–Mg bridging hydrogen atom was located from a ΔF map and refined freely.

Crystal Data for $C_{84}H_{115}CoMg_2N_4O_2$, M =1320.34, monoclinic, space group $P2_1/c$ (no. 14), a = 13.9878(4) Å, b = 13.5935(8) Å, c = 41.687(3) Å, β = 92.375(5)°, V = 7919.7(7) Å^3, Z = 4, ρ_{calc}g/cm^3 = 1.107, μ(CuKα) = 2.199 mm^{-1}, T = 173(2), Orange tabular needless, F^2 refinement, R_1(obs) = 0.1004, wR_2(all) = 0.2711, 15158 independent observed reflections (R_{int} = 0.0786), 8175 independent measured reflections [$|F_o| > 4\sigma(|F_o|)$, $2\theta_{full}$ = 147.436], 917 parameters. CCDC no. 2116007.

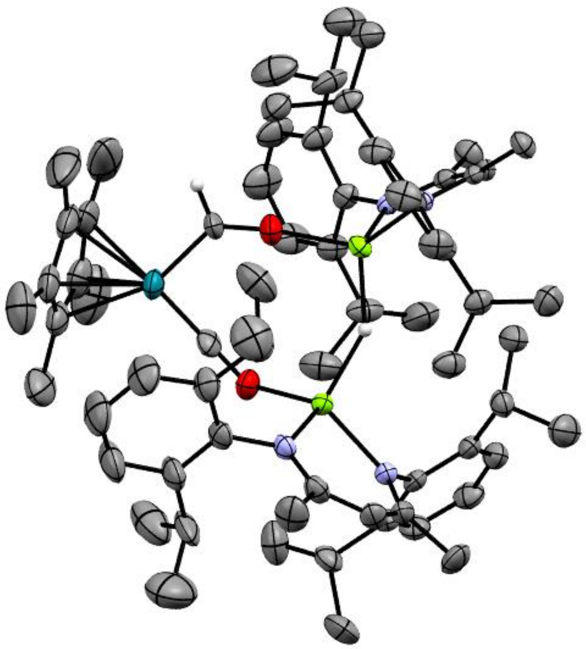

Figure S7. The X-ray crystal structure of **3g**. Most hydrogen atoms and included hexane molecules omitted for clarity.

3g was found to crystallise in the space group $P2_1/n$ space group, with two included hexane molecules in the aystmmetric unit. The C12-, C24-, and C27-based *iso*-propyl groups, and the C91-based included hexane solvent molecule, in the structure of **3g** were all found to be disordered and in each case two orientations were identified, of *ca*. 61:39, 69:31, 80:20 and 65:35% occupancy respectively. The geometries of each pair of orientations were optimised, the thermal parameters of adjacent atoms were restrained to be similar, and only the non-hydrogen atoms of the major occupancy orientations were refined anisotropically (those of the minor occupancy orientations were refined isotropically). The C61-based C(H)–O unit and the C60-based C=O moiety were also found to be disordered. Two orientations were identified of *ca*. 59 and 41% occupancy, with the minor occupancy orientation representing a "flipping" of the C(H)–O and C=O groups. (As the structure was presumed to always be a "mixed" C(H)–O/C=O species, the disorders of the two groups were treated as linked.) The thermal parameters of adjacent atoms were restrained to be similar, and only the non-hydrogen atoms of the major occupancy orientation were refined anisotropically (those of the minor occupancy orientation were refined isotropically). The Mg–H–Mg bridging hydrogen atom was located from a ΔF map and refined freely.

Crystal Data for $C_{79}H_{120}Mg_2N_4O_2Rh$, *M* =1309.31, monoclinic, space group $P2_1/n$ (no. 14), *a* = 14.0176(4) Å, *b* = 13.9077(5) Å, *c* = 40.2691(10) Å, *β* = 99.749(3)°, *V* = 7737.2(4) Å^3, *Z* = 4, ρ_{calc}g/cm^3 = 1.124, μ(MoKα) = 0.282 mm^{-1}, *T* = 173(2), Yellow blockss, F^2 refinement, R_1(obs) = 0.0443, wR_2(all) = 0.1068, 15686 independent observed reflections (R_{int} = 0.0313), 11939 independent measured reflections [$|F_o| > 4\sigma(|F_o|)$, $2\theta_{full}$ = 56.78], 892 parameters. CCDC no. 2116008.

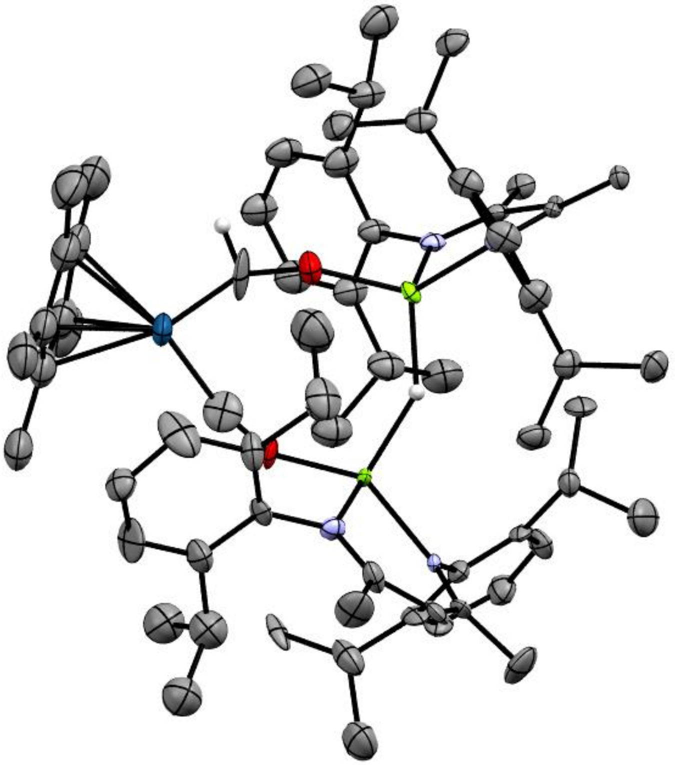

Figure S8. The X-ray crystal structure of **3h**. Most hydrogen atoms and included hexane molecules omitted for clarity.

3h was found to crystallise in the space group $P2_1/n$ space group, with two included hexane molecules in the aystmmetric unit. Though the original diffraction images for the crystal of **3h** that was studied looked to be of high quality, reciprocal space analysis of the data set clearly showed the crystal to be badly twinned. Initial indexing found a *C*-face centred orthorhombic unit cell using only *ca*. 48% of the observed spots with a very broad distribution histogram along the *b* axis. Transforming this to the related half-volume primitive monoclinic unit cell gave the same unit cell as seen for the rhodium analogue **3g** whilst using *ca*. 72% of the observed spots with a broad distribution histogram along the *c* axis (though narrower than seen for the *b* axis for the *C*-face centred orthorhombic unit cell). The best twinning model that could be found only raises the indexing to *ca*. 82% by adding one extra lattice orientation in a *ca*. 77:23 ratio, with the two lattices related by the approximate twin law [–1.00 0.00 0.00 0.00 –1.00 0.00 0.98 0.00 1.01]. Unfortunately, the structure arising from this best model is not great, with significant disorder and somewhat high final *R*-factors. The gross structure is reliable, however, showing the identities of the major components present, especially since this iridium structure is isomorphous with its rhodium analogue **3g**.

The C12-, C24-, and C27-based *iso*-propyl groups, and the C91-based included hexane solvent molecule were all found to be disordered and in each case two orientations were identified, of *ca*. 59:41, 62:38, 51:49 and 66:34% occupancy respectively. The geometries of each pair of orientations were optimised, the thermal parameters of adjacent atoms were restrained to be similar, and only the non-hydrogen atoms of the major occupancy orientations were refined anisotropically (those of the minor occupancy orientations were refined isotropically). The C61-based C(H)–O unit and the C60-based C=O moiety were also found to be disordered. Two orientations were identified of *ca*. 74 and 26% occupancy, with the minor occupancy orientation representing a "flipping" of the C(H)–O and C=O groups. (As the structure was presumed to always be a "mixed" C(H)–O/C=O species, the disorders of the two groups were treated as linked.) The thermal parameters of adjacent atoms were restrained to be similar, and only the non-hydrogen atoms of the major occupancy orientation were refined anisotropically (those of the minor occupancy orientation were refined isotropically). The Mg–H–Mg bridging hydrogen atom was located from a Δ*F* map and refined freely. Numerous thermal parameter restraints had to be applied to handle poor thermal ellipsoid shapes, likely a result of the poorly resolved twinning.

Crystal Data for $C_{79}H_{120}IrMg_2N_4O_2$, *M* =1398.60, monoclinic, space group $P2_1/n$ (no. 14), *a* = 14.0273(6) Å, *b* = 13.9062(6) Å, *c* = 40.2048(14) Å, *β* = 99.779(4)°, *V* = 7728.7(5) Å^3, *Z* = 4, ρ_{calc}g/cm^3 = 1.202, μ(MoKα) = 1.788 mm^{-1}, *T* = 173(2), Pale yellow tablets, F^2 refinement, R_1(obs) = 0.1271, wR_2(all) = 0.3310, 16394 independent observed reflections (R_{int} = 0.0663), 11538 independent measured reflections [$|F_o| > 4\sigma(|F_o|)$, $2\theta_{full}$ = 56.33], 890 parameters. CCDC no. 2116009.

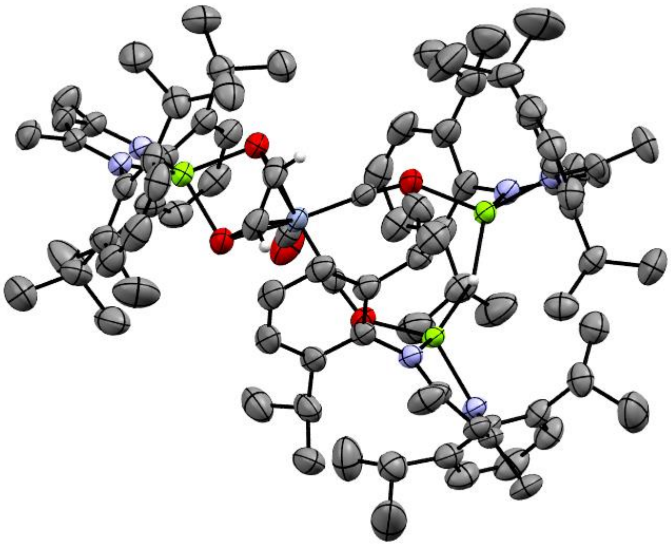

Figure S9. The X-ray crystal structure of **4a**. Most hydrogen atoms and included hexane molecules omitted for clarity.

The C101- and C131-based included hexane solvent molecules in the structure of **4a** were found to be disordered, and in each case three orientations were identified, of *ca*. 39:32:29 and 47:37:16% occupancy respectively. The geometries of each set of orientations were optimised, the thermal parameters of adjacent atoms were restrained to be similar, and all of the atoms were refined isotropically. The Mg1–H1–Mg2 bridging hydrogen atom was located from a Δ*F* map and refined freely. The C–H hydrogen atoms on C63 and C64 were initially located from Δ*F* maps, and then included in calculated positions.

Crystal Data for $C_{104}H_{154}CrMg_3N_6O_5$, *M* =1693.25, monoclinic, space group $P2_1/c$ (no. 14), a = 19.4330(2) Å, b = 17.40489(18) Å, c = 30.8931(4) Å, β = 96.4115(12)°, V = 10383.6(2) Å^3, Z = 4, ρ_{calc}g/cm^3 = 1.083, μ(CuKα) = 1.469 mm^{-1}, T = 173(2), Orange blocky needless, F^2 refinement, R_1(obs) = 0.0570, wR_2(all) = 0.1667, 19933 independent observed reflections (R_{int} = 0.0445), 13294 independent measured reflections [$|F_o| > 4\sigma(|F_o|)$, $2\theta_{full}$ = 146.93], 1148 parameters. CCDC no. 2116010.

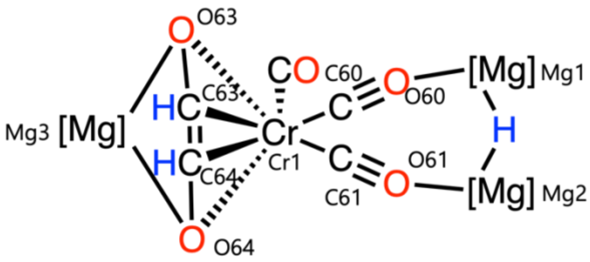

Figure S10. Key bond lengths for **4a**.

Bond	Bond length (Å)	Bond	Bond length (Å)	Bond	Bond length (Å)
Cr1-C63	2.109(3)	Cr1-C64	2.104 (3)	C63-C64	1.369(5)
Cr1-O63	2.164(2)	Cr1-O64	2.115(2)	C63-O63	1.355(4)
C64-O64	1.361(4)	O63-Mg3	1.986(2)	O64-Mg3	1.992(2)
Cr1-C60	1.766(3)	Cr1-C61	1.758(3)	C60-O60	1.197(3)
C61-O61	1.211(3)	O60-Mg1	1.983(2)	O61-Mg2	1.993(2)

Table **S1**. Selected structural data for **4a**.

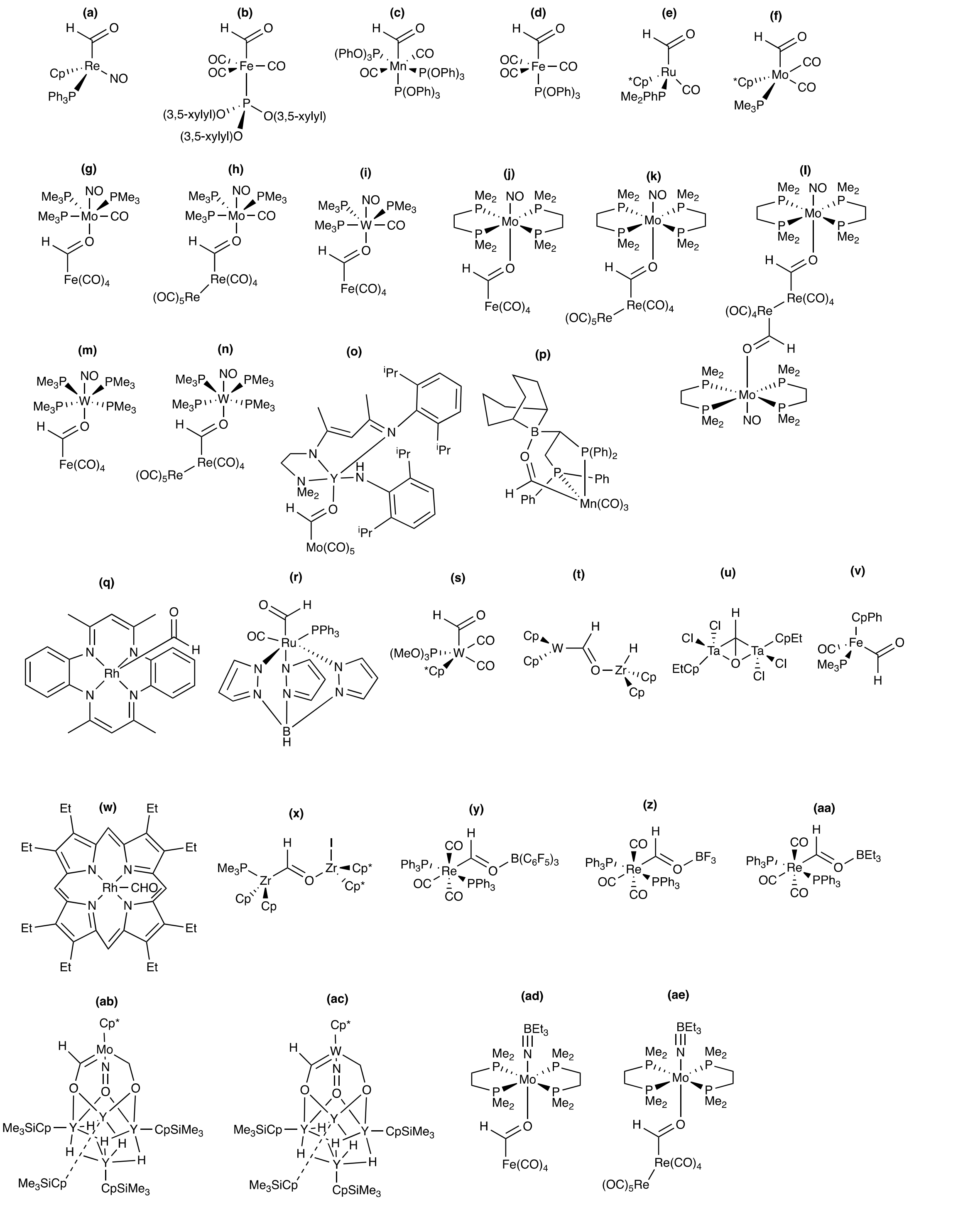

Figure S11. Reported structurally characterised transition metal formyl complexes (Cp = cyclopentadienyl, Cp* = pentamethylcyclopentadienyl, CpPh = pentaphenylcyclopentadienyl, CpEt = ethyltetramethylcyclopentadienyl, $CpSiMe_3$ = trimethylsilyltetramethylcyclopentadienyl).

Complex	M–C (Å)[a, b]	C–O (Å)[a, b]	M–C–O (°)[a]	M–C–H (°)[a]	CONQUEST	Identifier[c]	Reference
**(a)**	2.0552	1.2207	128.08	119.20	FMCPRE	1157892	[3]
**(b)**	1.9484	1.1658	133.73	127.62	BIRVON	1111558	[4]
**(c)**	2.04(1)	1.21(1)	126.5(7)	131.3	COLZEI	1129261	[5]
**(d)**	1.976(3)	1.166(4)	133.0(3)	117(3)	CUBGEL	1132109	[4]
**(e)**	2.039(5)	1.099(5)	140.3(3)	103(3)	CIMXAX	1125748	[6]
**(f)**	2.156(9)	1.21(1)	138.1(8)	106.5	JAXSEG	1183427	[7]
**(g)**	1.917(3)	1.188(3)	130.7(2)	119(2)	WITZUU	137761	[8]
**(h)**	2.102(7)	1.264(9)	127.5(5)	113(4)	WIVBAE	137762	[8]
**(i)**	2.05(1)	1.17(1)	140.8(8)	109.6	QIRMUZ	157338	[9]
**(j)**	1.95	1.12	136.4	111.8	ELEGEH	198215	[10]
**(k)**	2.18(1)	0.91(2)	169(1)	95.0	ELEGIL	198216	[10]
**(l)**	2.140(8)	1.23(1)	127.6(6)	116.2	ELEGOR	198217	[10]
	2.132(8)	1.25(1)	127.9(6)	116.2		198217	
**(m)**	1.945(3)	1.210(4)	129.6(3)	115.2	FIHBEE	250879	[11]
**(n)**	2.10(1)	1.13(1)	141(1)	110.0	FIHBII	250880	[11]
**(o)**	2.136(6)	1.248(8)	131.4(5)	114.3	QEJGAP	897701	[12]
**(p)**	1.988(1)	1.265(2)	135.6(1)	115.4(9)	PEZZUR	929476	[13]
**(q)**	1.931(4)	1.179(4)	128.4(2)	110(2)	BOGPAQ	1002882	[14]
**(r)**	2.01(1)	1.23(2)	140.7(9)	109.5	YAYGEN	891753	[15]
**(s)**	2.11(1)	1.17(2)	144(1)	107.8	ASIPAW	2069826	[16]
**(t)**	No coord on CCDC				CPWCZR	1131515	[17]
**(u)**	2.0848	2.0940	69.35	n/a	FRMHTA	1160279	[18]
**(v)**	1.82(2)	1.10(8)	150(3)	n/a	YECHIX	1300556	[19]
**(w)**	No coord on CCDC				BEGDIA	1107890	[20]
**(x)**	2.123(5)	1.37(1)	139.6(5)	108(4)	COPVIM	1129701	[21]
**(y)**	2.125(5)	1.282(6)	126.9(3)	116.5	CUQROW	685990	[22]
**(z)**	2.096(3)	1.270(4)	125.8(2)	117.1	CUQSAJ	762056	[22]
**(aa)**	2.150(3)	1.257(3)	122.6(2)	118.7	OLUSIY	770577	[23]
**(ab)**	1.924(9)	1.42(1)	128.4(7)	n/a	LUMTIX	738452	[24]
**(ac)**	1.89(1)	1.37(1)	133.6(8)	113.2	LUMTOD	738453	[24]
**(ad)**	1.933(4)	1.220(3)	132.0(2)	114.0	SECRAU	280782	[25]
**(ae)**	2.137(4)	1.250(4)	130.6(3)	114.7	SECREY	280783	[25]

Table S2. Selected structural parameters for reported transition metal formyl complexes **3**. [a] Metal-carbon formyl unit. [b] average bond length, provided with pooled estimated standard deviation (ESDs) in parentheses where possible. [c] CCDC Deposition Number.

4. IR Spectroscopy

Complex	M–C≡O–Mg (cm^{-1}) [bridging]	M–C≡O (cm^{-1}) [terminal]
**3a**	1776 (s)	1943 (s), 1947 (m), 1987 (s)
**3b**	1769 (s)	1936 (s), 1942 (s), 1987 (w), 2067 (w)
**3c**	1784 (s)	1903 (s), 1951 (s), 1981 (w)
**3d**	1674 (s)	1916 (s)
**3e**	1733 (m)	1949 (m)
**3f**	1791 (s)	-
**3g**	1784 (s)	-
**3h**	1785 (s)	-
**4a**	1674 (s), 1744 (s)	1982 (s)

Table S3. Selected infrared spectroscopy data for complexes **3** and **4**.

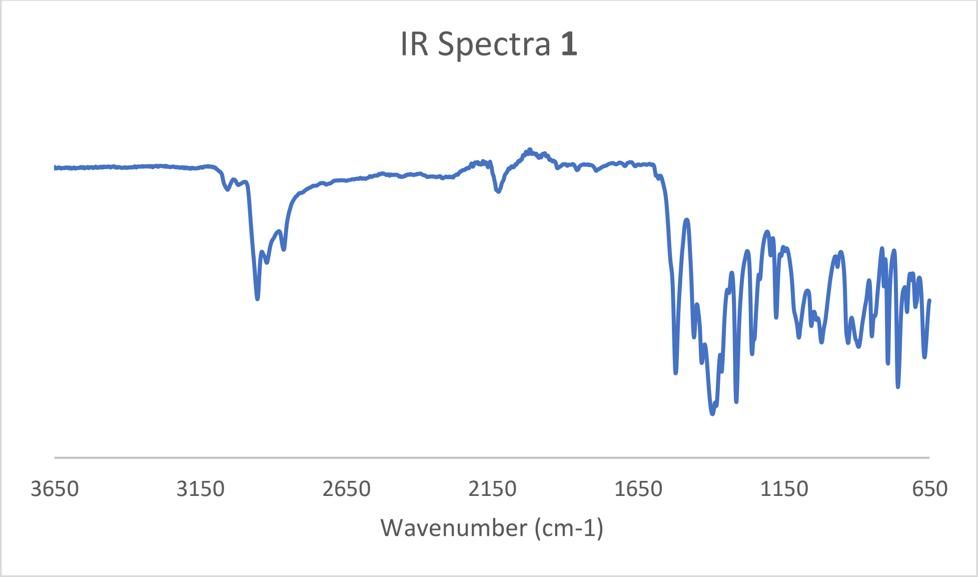

Figure S12. IR Spectra for **1.**

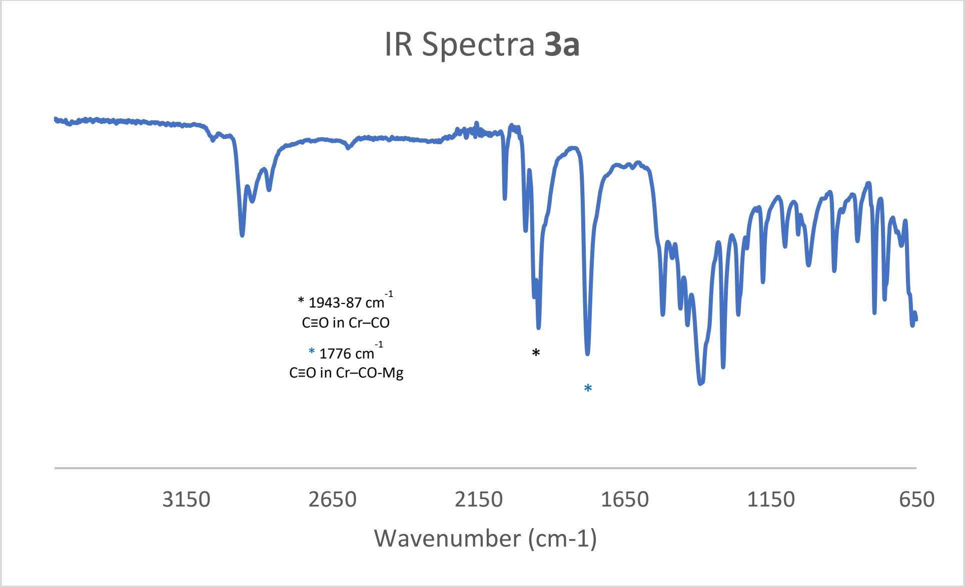

Figure S13. IR Spectra for **3a.**

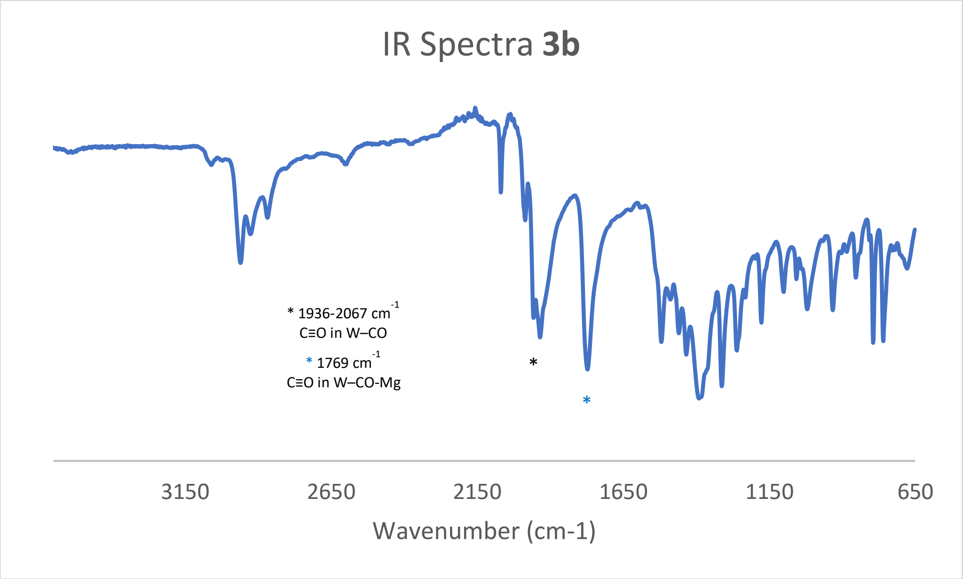

Figure S14. IR Spectra for **3b.**

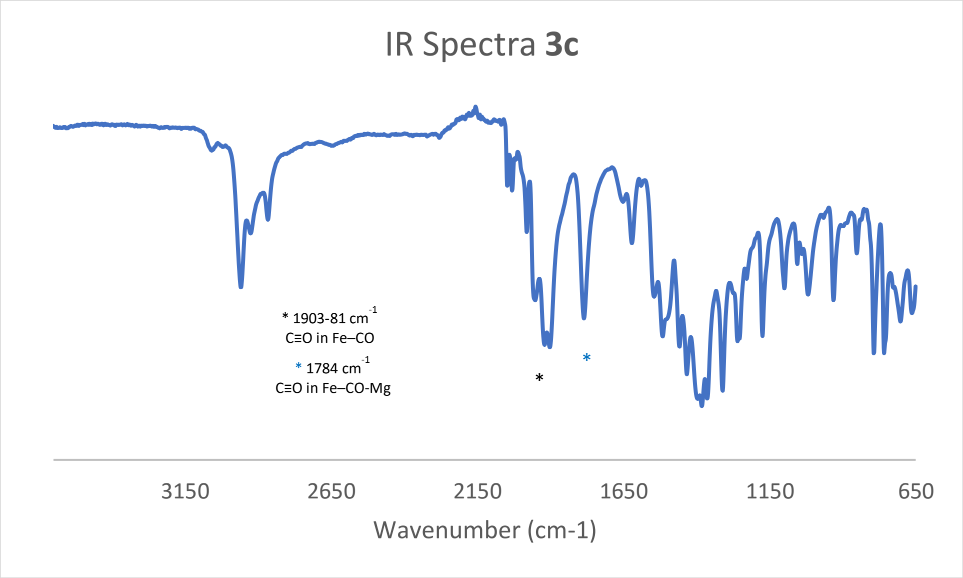

Figure S15. IR Spectra for **3c.**

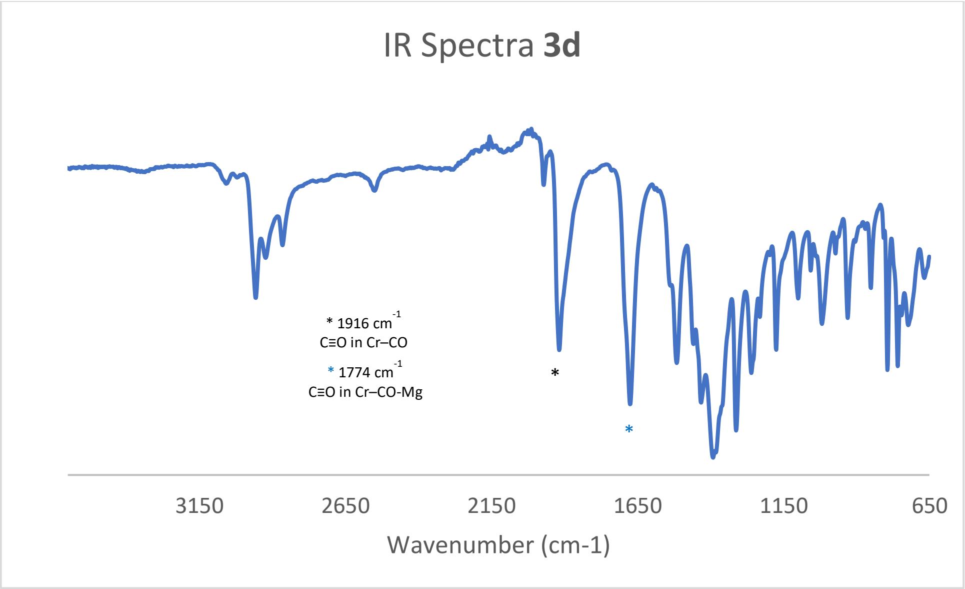

Figure S16. IR Spectra for **3d.**

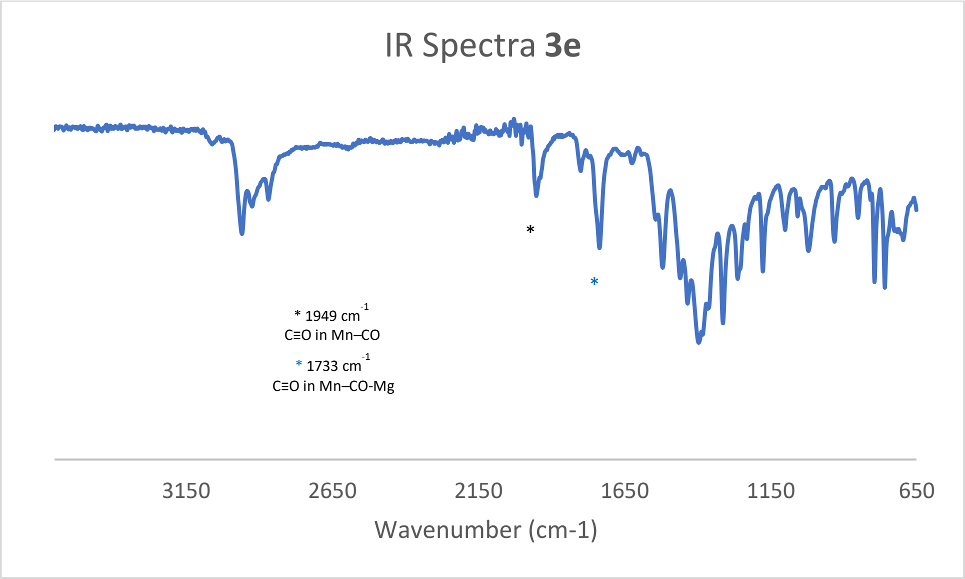

Figure S17. IR Spectra for **3e.**

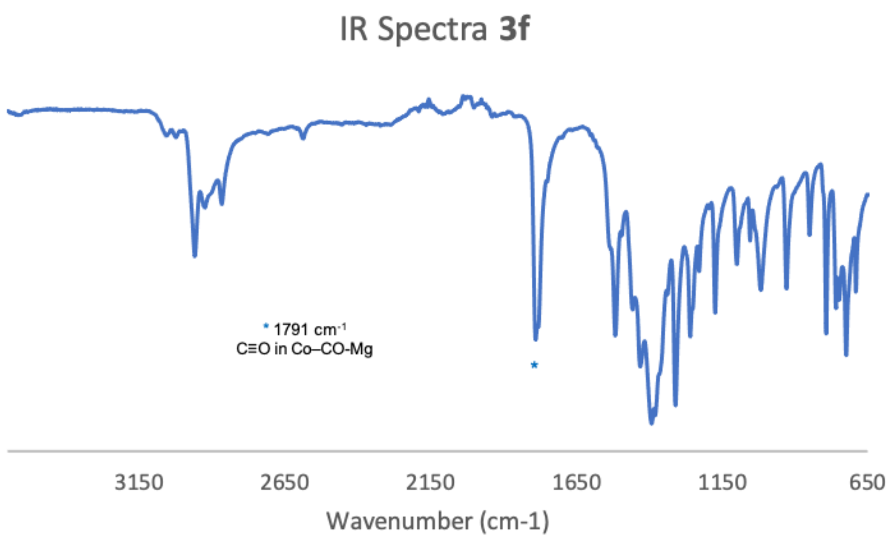

Figure S18. IR Spectra for **3f.**

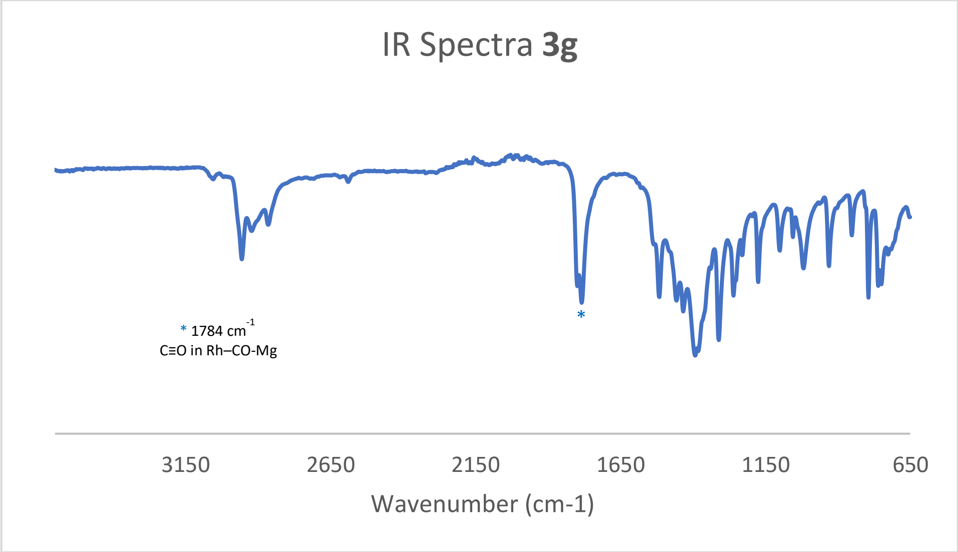

Figure S19. IR Spectra for **3g.**

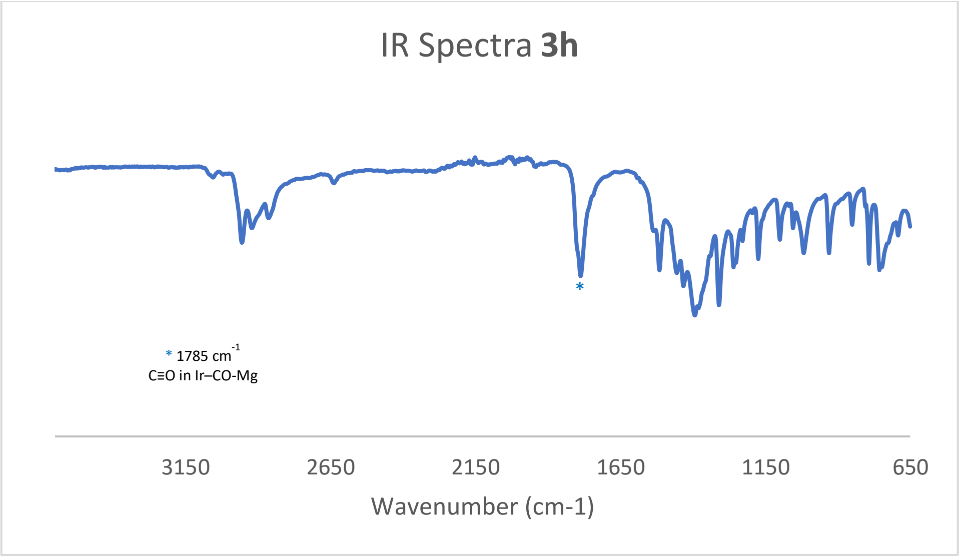

Figure S20. IR Spectra for **3h.**

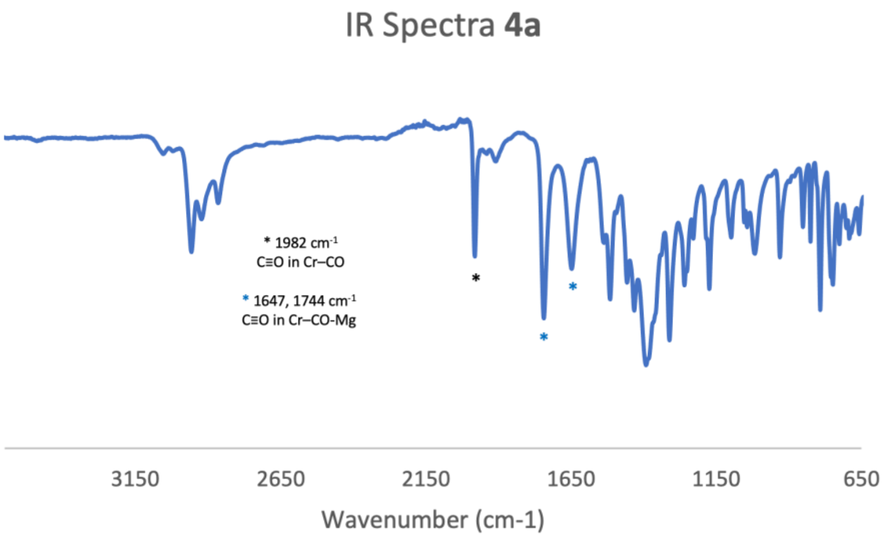

Figure S21. IR Spectra for **4a.**

5. Stability Studies

Synthetic methodology:

In a N_2 filled glovebox an approximately 0.1 M solution of **3** in C_6D_6 was transferred to a J-Young NMR tube fitted with a 0.1 M ferrocene external reference and the solution immediately interrogated by ^{1}H NMR spectroscopy. The stability of **3** was quantified through repeat runs and the normalised ratio of formyl proton to ferrocene insert tabulated.

Complex	k_{obs} (hour^{-1})	$t_{1/2}$ (hours)
**3a**	0.3328	2
**3a**[a]	0.513	1
**3b**	0.3159	2
**3c**	0.0121	57
**3d**	0.0051	136
**3e**	0.0031	224
**3f**	0.0021	330
**3g**	0.0032	217
**3h**	0.0013	533

Table S4. Observed rate constant (k_{obs}) and half-life ($t_{1/2}$) for the decomposition of complexes **3** in C_6D_6 solution under nitrogen at 22 °C. [a] Under 1 atmosphere of carbon monoxide at 22 °C.

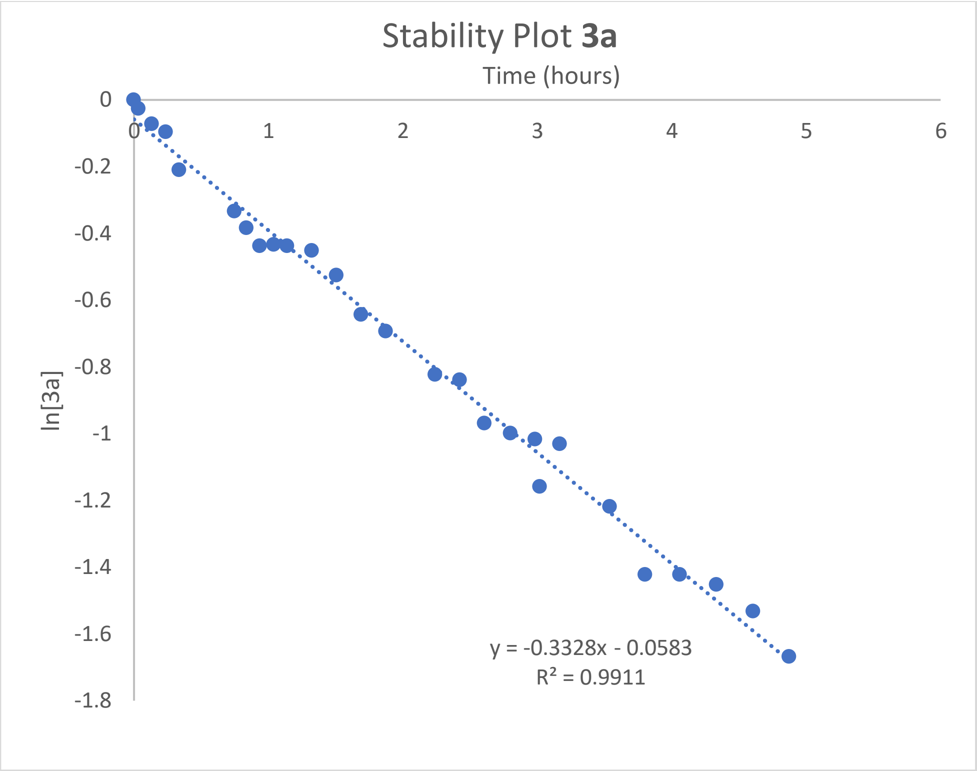

Figure S22. Stability plot for the decomposition of **3a** at room temperature (C_6D_6 solution, under N_2)

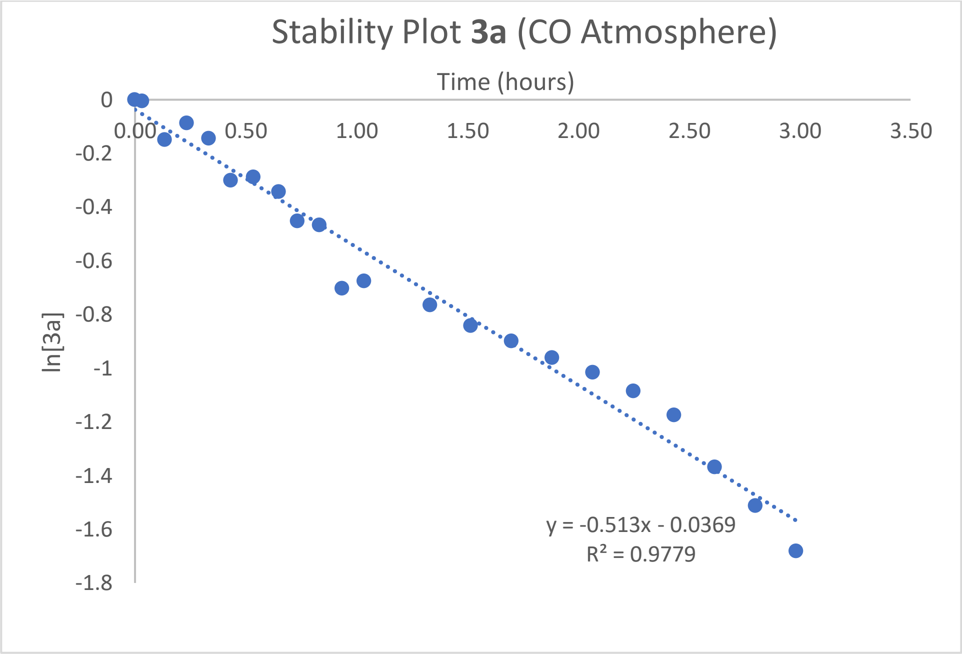

Figure S23. Stability plot for the decomposition of **3a** at room temperature (C_6D_6 solution, under CO)

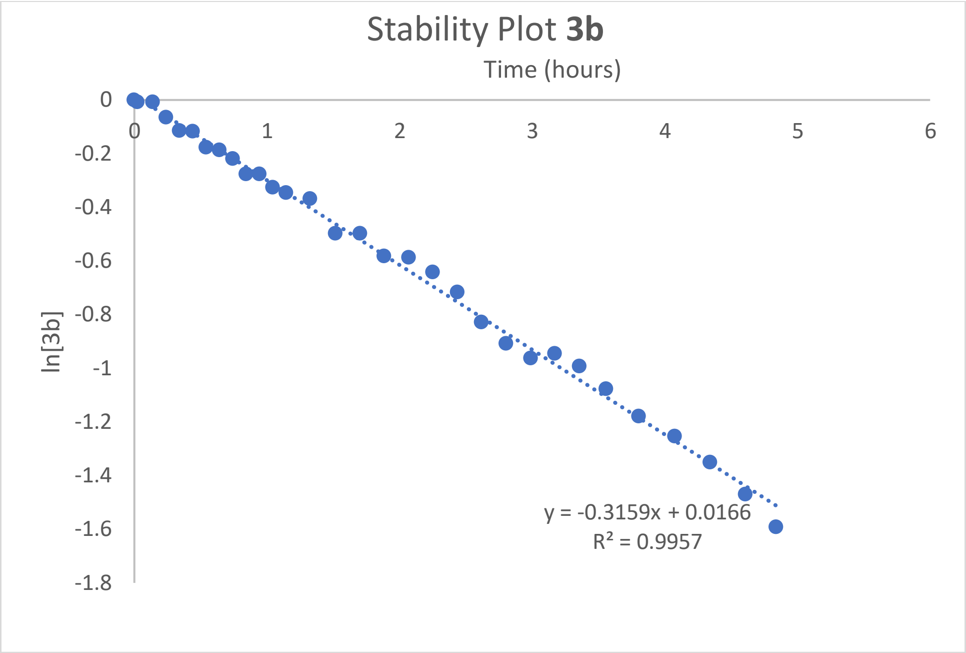

Figure S24. Stability plot for the decomposition of **3b** at room temperature (C_6D_6 solution, under N_2)

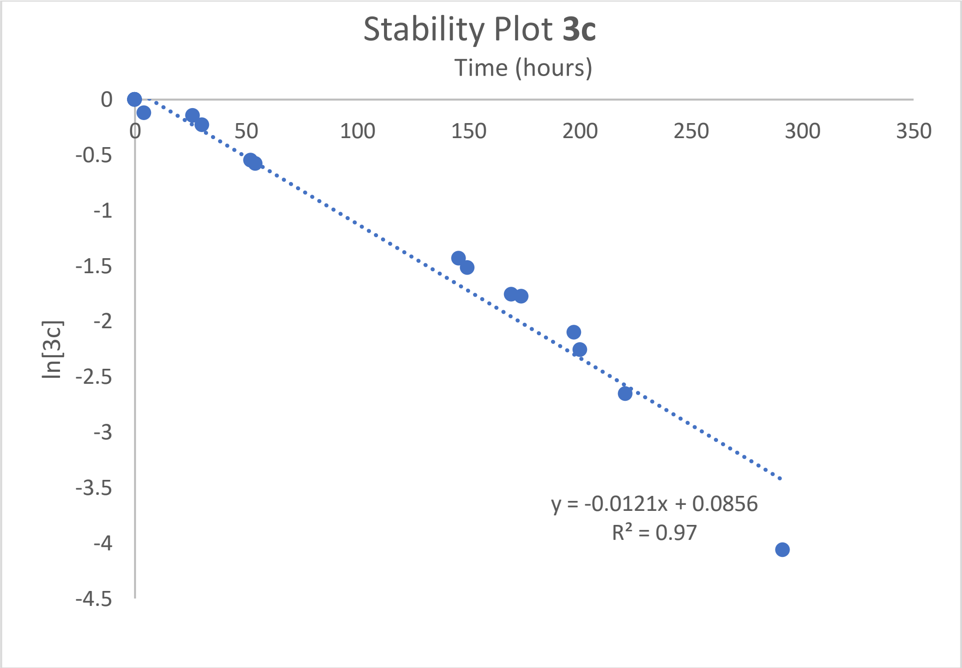

Figure S25. Stability plot for the decomposition of **3c** at room temperature (C_6D_6 solution, under N_2)

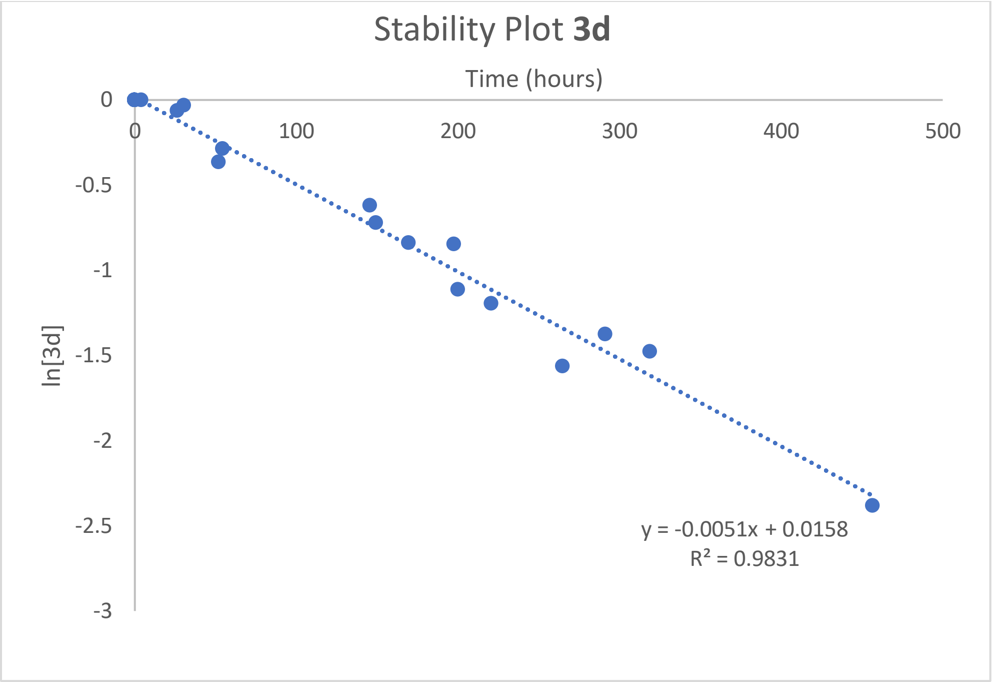

Figure S26. Stability plot for the decomposition of **3d** at room temperature (C_6D_6 solution, under N_2)

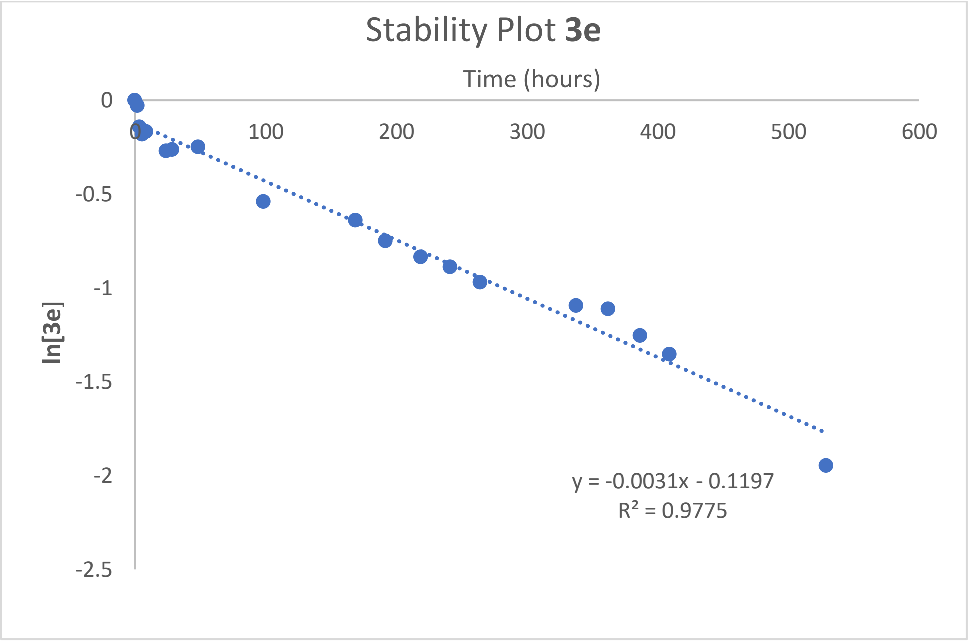

Figure S27. Stability plot for the decomposition of **3e** at room temperature (C_6D_6 solution, under N_2)

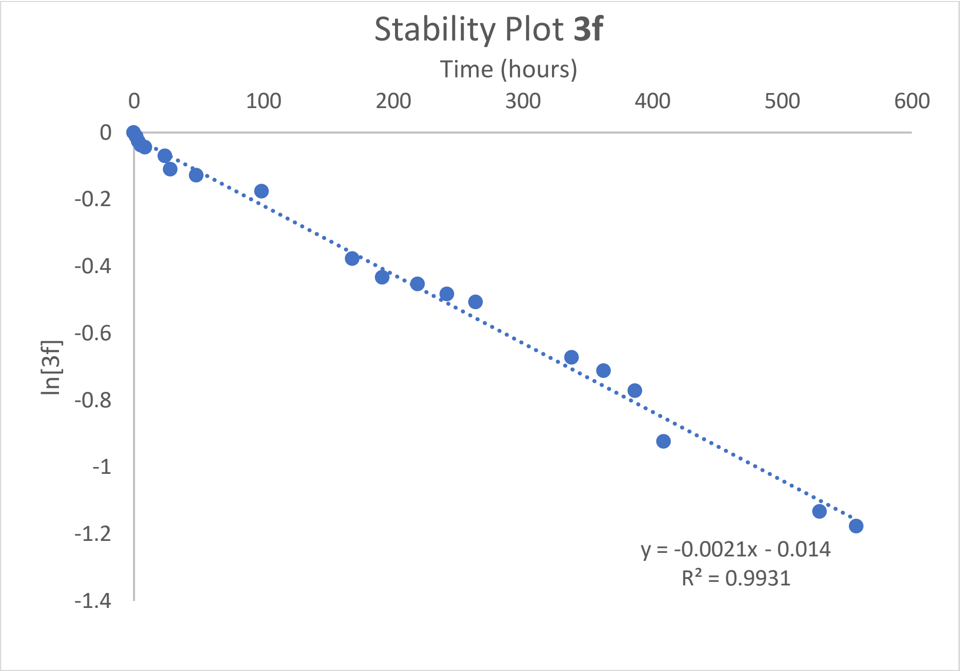

Figure S28. Stability plot for the decomposition of **3f** at room temperature (C_6D_6 solution, under N_2)

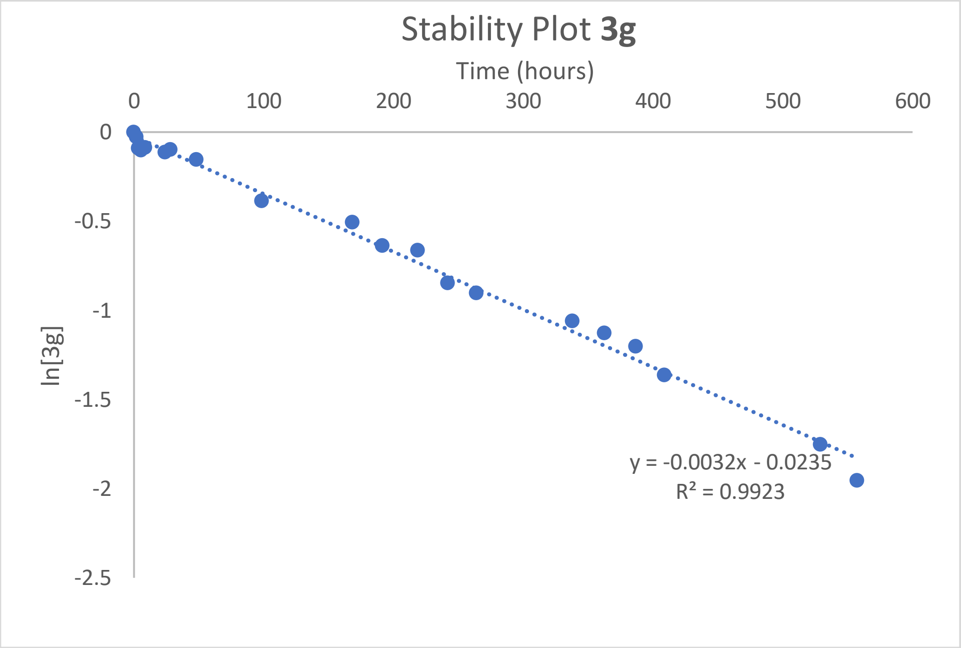

Figure S29. Stability plot for the decomposition of **3g** at room temperature (C_6D_6 solution, under N_2)

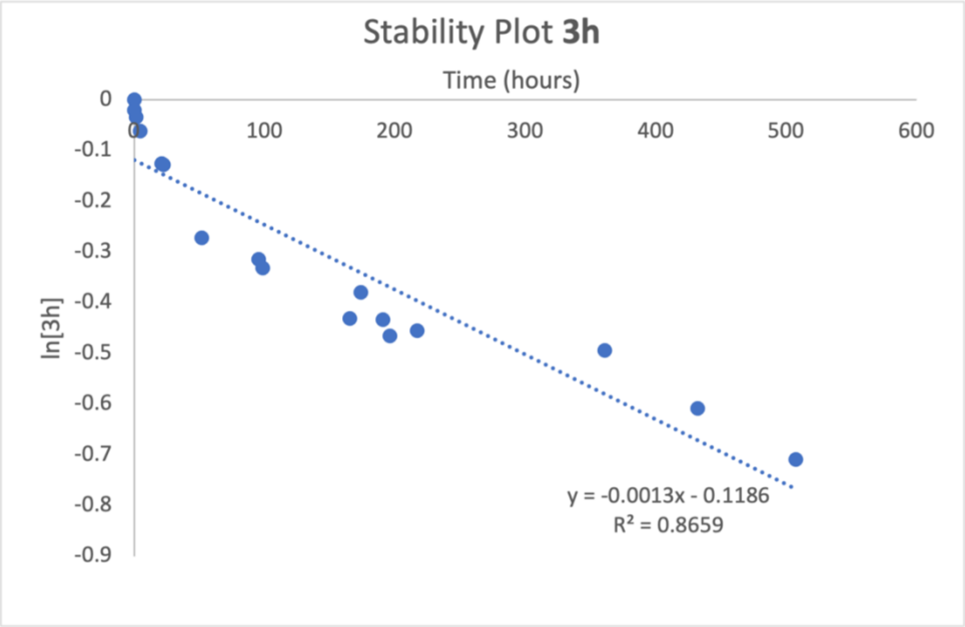

Figure S30. Stability plot for the decomposition of **3h** at room temperature (C_6D_6 solution, under N_2)

6. **Computational Methods**

Density Functional Theory (DFT) calculations were run using Gaussian 09 (Revision D.01)[26] using the ωB97X-D[27] functional and an ultrafine integrations grid (keyword int=ultrafine). Metal atoms (Mg, Cr, Mn, Fe, Co, Rh, W, Ir) were described with Stuggart SDDAll RECPs and associated basis sets, while a hybrid basis set was used for the other atoms: 6-31g**(C, H)/ 6-311+g*(N, O). The level of theory used has previously been benchmarked in our group and shown to accurately reproduce the experimental results.[28–31] Geometry optimisations were performed without symmetry constraints. Frequency analyses for all stationary points were performed to confirm ther nature of the structures as either minima (no imaginary frequency) or transition states (only one imaginary frequency). Single point solvent corrections (toluene, epsilon = 2.3741) were applied using the polarized continuum model (PCM) to free energies.[32] Intrinsic Reaction Coordinate (IRC) calculations followed by full geometry optimisations on final points were used to connect transition states and minima located on the Potential Energy Surface. A full energy profile was constructed (calculated at 298.15 K, 1 atm). The graphical user interface used to visualise the various properties of complexes **3/4**, and to construct the relevant fragments for ETS-NOCV calcualtions, was GaussView 5.0.9.[33]

ETS-NOCV[34] calculations were performed using DFT as implemented in Orca 4.2.1.[35,36] Optimised geometries of complexes **3** from the Gaussian 09 calculations detailed above were used. Single-point calculations were performed using the ωB97X-D3[37] functional. The def2-tzvpp basis set was used for all atoms. Graphical surface representations shown below were plotted using Avogadro 1.2.0.

Natural Bond Orbital analysis was carried outin NBO 6.0.[38,39] A full NBO analysis for **3a-h** was carried out and the relevant NPA charges and Wiberg Bond Indices tabulated (Table S5-6).

QTAIM calculations were performed using the AIMAll sotware.[40,41]

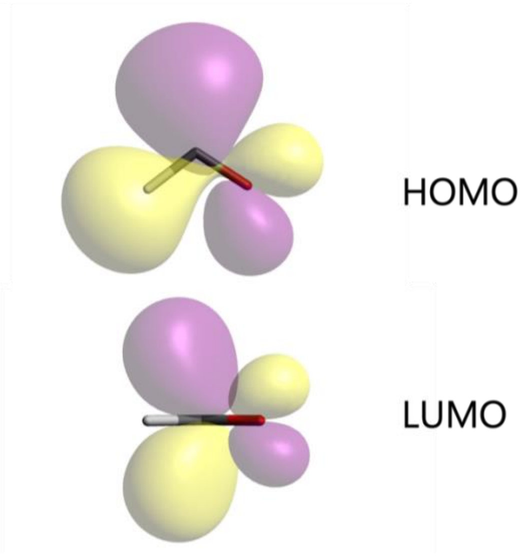

Figure S31. Selected Calculate Molecular Orbitals for the {CHO}$^{-}$ Fragment.

	Bond							
	TM–C(formyl)	C–(formyl)	O(formyl) –Mg	Mg–H	H–Mg	Mg–O (isocarb)	O–C (isocarb)	C(isocarb) –TM
**3a**	0.55	1.52	0.052	0.13	0.17	0.045	1.79	0.97
$[Cr(CO)_5(CHO)]^-$	0.46	1.80	-	-	-	-	-	-
**3-Mo**	0.59	1.53	0.054	0.14	0.17	0.047	1.80	1.06
$[Mo(CO)_5(CHO)]^-$	0.48	1.80	-	-	-	-	-	-
**3b**	0.60	1.53	0.054	0.14	0.17	0.047	1.78	1.10
$[W(CO)_5(CHO)]^-$	0.49	1.80	-	-	-	-	-	-
**3c**	0.57	1.52	0.055	0.13	0.17	0.050	1.74	0.84
**3d**	0.87	1.44	0.057	0.14	0.16	0.049	1.64	1.39
**3e**	0.77	1.50	0.06	0.15	0.16	0.049	1.67	1.28
**3f**	0.79	1.42	0.06	0.14	0.17	0.052	1.67	1.14
**3g**	0.85	1.44	0.058	0.14	0.17	0.051	1.67	1.25
**3h**	0.95	1.40	0.058	0.14	0.17	0.052	1.62	1.42

Table S5. Summary of Wiberg bond indices for complexes **3a-h**.

	Atom							
	TM	C (formyl)	O (formyl)	Mg	H	Mg	O	C
**3a**	-1.29	0.27	-0.86	1.78	-0.80	1.75	-0.71	0.67
$[Cr(CO)_5(CHO)]^-$	-1.28	0.21	-0.63	-	-	-	-	-
**3-Mo**	-1.08	0.23	-0.86	1.76	-0.80	1.75	-0.69	0.68
$[Mo(CO)_5(CHO)]^-$	-1.10	0.16	-0.64	-	-	-	-	-
**3b**	-0.86	0.19	-0.86	1.76	-0.80	1.75	-0.69	0.63
$[W(CO)_5(CHO)]^-$	-0.89	0.12	-0.64	-	-	-	-	-
**3c**	-0.46	0.25	-0.85	1.77	-0.80	1.75	-0.74	0.53
**3d**	-0.66	0.30	-0.91	1.77	-0.80	1.75	-0.79	0.71
**3e**	-0.42	0.31	-0.87	1.77	-0.80	1.75	-0.77	0.70
**3f**	0.21	0.21	-0.91	1.77	-0.79	1.74	-0.77	0.57
**3g**	0.17	0.23	-0.89	1.77	-0.79	1.75	-0.76	0.53
**3h**	0.28	0.18	-0.90	1.77	-0.79	1.75	-0.77	0.48

Table S6. Summary of Natural Population Analysis (NPA) charges for complexes **3** and anionic formyl complexes.

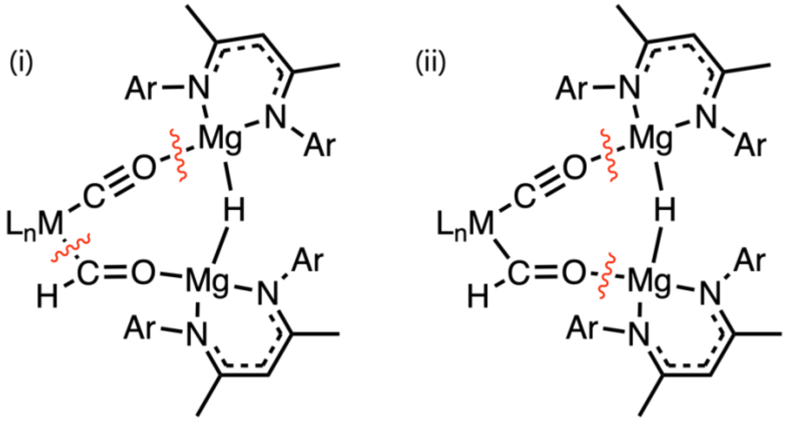

Figure S32. Splitting of fragments for ETS-NOCV calculations for complexes **3**.

	ΔE_{ORB}	$\Delta\rho1$	$\Delta\rho2$	$\Delta\rho3$
**3a**	-92.2	-53.9 (58)	-11.7 (13)	-8.3 (9)
**3b**	-191.7	-147.8 (77)	-11.7 (6)	-10.8 (5)
**3c**	-115.5	-71.6 (62)	-14.1 (12)	-10.3 (9)
**3d**	-107.6	-52.1 (48)	-18.8 (17)	-12.8 (12)
**3e**	-107.7	-56.4 (52)	-16.5 (15)	-12.9 (12)
**3f**	-108.6	-50.6 (47)	-23.8 (22)	-10.5 (10)
**3g**	-155.6	-90.0 (58)	-22.9 (15)	-12.2 (8)
**3h**	-579.1	-452.6 (78)	-82.2 (14)	-12.3 (2)

Table S7. Summary of ETS-NOCV data for **3** with (i) splitting. All values in kcal mol^{-1}. Percentage contribution of $\Delta\rho_x$ to ΔE_{ORB} included in brackets.

	ΔE_{ORB}	$\Delta\rho1$	$\Delta\rho2$	$\Delta\rho3$
**3a**	-53.1	-9.2	-7.9	-6.3
**3b**	-54.1	-7.8	-7.7	-6.7
**3c**	-53.2	-9.5	-8.4	-5.7
**3d**	-67.1	-8.8	-8.8	-7.2
**3e**	-61.3	-10.8	-8.3	-6.0
**3f**	-63.2	-9.0	-8.2	-6.4
**3g**	-62.6	-8.5	-8.2	-6.7
**3h**	-66.2	-9.2	-9.0	-7.4

Table S8. Summary of ETS-NOCV data for **3** with (ii) splitting. All values in kcal mol^{-1}.

	Atom							
	TM	C (formyl)	O (formyl)	Mg	H	Mg	O	C
**3a**	1.13	0.68	-1.37	1.72	-0.79	1.72	-1.37	0.89
**3b**	1.78	0.61	-1.39	1.72	-0.79	1.71	-1.37	0.75
**3c**	0.73	0.75	-1.37	1.72	-0.79	1.71	-1.38	0.87
**3d**	1.06	0.64	-1.39	1.72	-0.79	1.72	-1.40	0.71
**3e**	0.93	0.72	-1.37	1.72	-0.79	1.72	-1.39	0.79
**3f**	0.63	0.69	-1.39	1.72	-0.79	1.71	-1.39	0.85
**3g**	0.50	0.75	-1.38	1.72	-0.79	1.71	-1.38	0.86
**3h**	0.57	0.72	-1.39	1.71	-0.79	1.71	-1.38	0.79

Table S9. QTAIM Charges for complexes **3a-h**.

	Bond ρ (ellipticity)								
	TM–C(formyl)	C–O (formyl)	O(formyl) –Mg	Mg–H	H–Mg	Mg–O (isocarb)	O–C (isocarb)	C(isocarb) –TM	RCP[a]
**3a**	0.098 (0.098)	0.36 (0.17)	0.044 (0.059)	0.034 (0.0099)	0.039 (0.0041)	0.032 (0.039)	0.43 (0.011)	0.14 (0.16)	0.018
**3b**	0.14 (0.11)	0.36 (0.17)	0.047 (0.078)	0.033(0.011)	0.039 (0.0040)	0.033 (0.039)	0.43 (0.013)	0.20 (0.019)	0.0011
**3c**	0.12 (0.079)	0.36 (0.15)	0.044 (0.071)	0.035 (0.011)	0.039 (0.0041)	0.034 (0.033)	0.42 (0.026)	0.18 (0.059)	0.0051
**3d**	0.13 (0.18)	0.35 (0.16)	0.050 (0.064)	0.034 (0.010)	0.038 (0.0055)	0.039 (0.030)	0.40 (0.020)	0.17 (0.22)	0.0044
**3e**	0.14 (0.17)	0.36 (0.14)	0.048 (0.046)	0.035 (0.0056)	0.037 (0.0053)	0.037 (0.035)	0.41 (0.024)	0.18 (0.13)	00040
**3f**	0.15 (0.16)	0.35 (0.13)	0.050 (0.053)	0.034 (0.016)	0.040 (0.0064)	0.037 (0.030)	0.41 (0.030)	0.20 (0.02)	0.052
**3g**	0.16 (0.18)	0.36 (0.12)	0.049 (0.055)	0.035 (0.014)	0.039 (0.0054)	0.037 (0.033)	0.41 (0.034)	0.19 (0.019)	0.0048
**3h**	0.17 (0.19)	0.35 (0.10)	0.050 (0.051)	0.035 (0.014)	0.040 (0.0052)	0.039 (0.035)	0.40 (0.039)	0.21 (0.043)	0.0046

Table S10. QTAIM data for bond-critical points for complexes **3a-h** .[a] Ring-Critical Point for 8-membered ring.

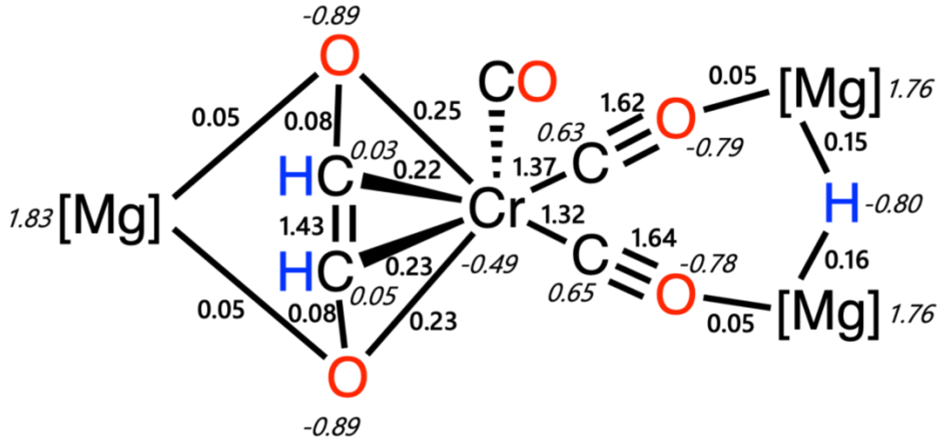

Figure S33. Select *NPA charges* and **Wiberg Bond Indices** for complex **4a**.

3 ΔE$_{ORB}$	Δρ1
**3a**<br>**-92.2 kcal mol^{-1}**	Δρ1<br>σC(H)O to Cr (3d)<br>-53.9 kcal mol^{-1}
**3b**<br>**-191.7 kcal mol^{-1}**	Δρ1<br>σC(H)O to W (5d)<br>-147.8 kcal mol^{-1}
**3c**<br>**-115.5 kcal mol^{-1}**	Δρ1<br>σC(H)O to Fe (3d)<br>-71.6 kcal mol^{-1}

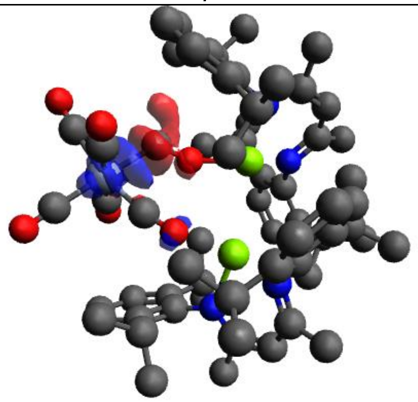

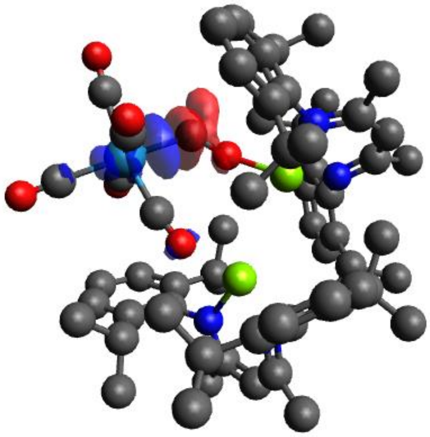

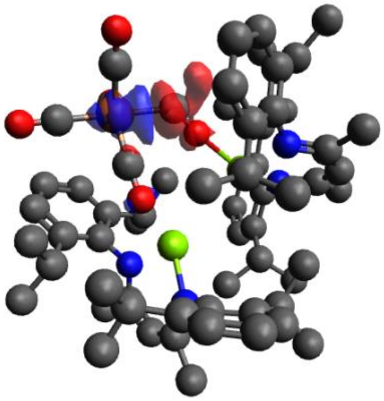

3d
-107.6 kcal mol^{-1}

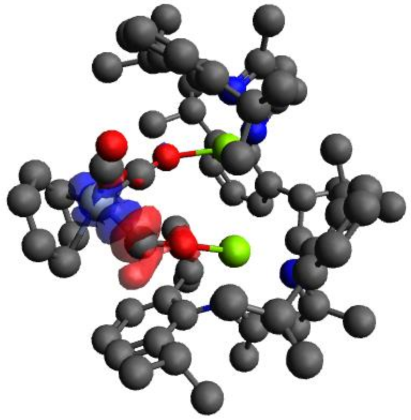

Δρ1
σC(H)O to Cr (3d)
-52.1 kcal mol^{-1}

3e
-107.7 kcal mol^{-1}

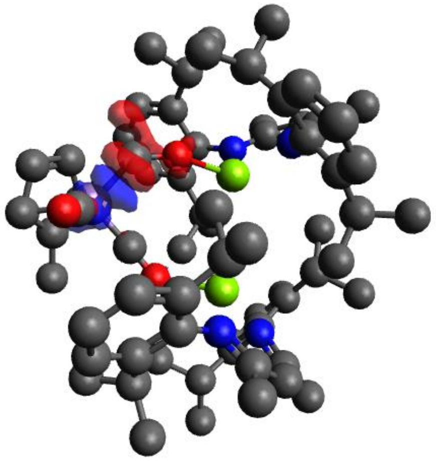

Δρ1
σC(H)O to Mn (3d)
-56.4 kcal mol^{-1}

3f
-108.6 kcal mol^{-1}

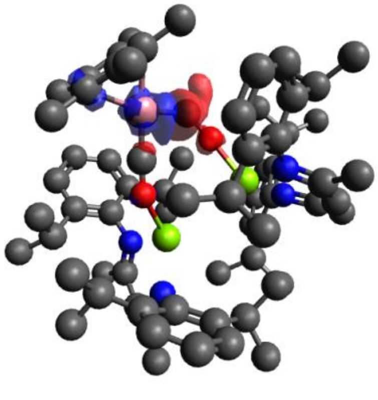

Δρ1
σC(H)O to Co (3d)
-50.6 kcal mol^{-1}

**3g**<br>**-155.6 kcal mol^{-1}**	Δρ1<br>σC(H)O to Rh (4d)<br>-90.0 kcal mol^{-1}
**3h**<br>**-579.1 kcal mol^{-1}**	Δρ1<br>σC(H)O to Ir (5d)<br>-452.6 kcal mol^{-1}

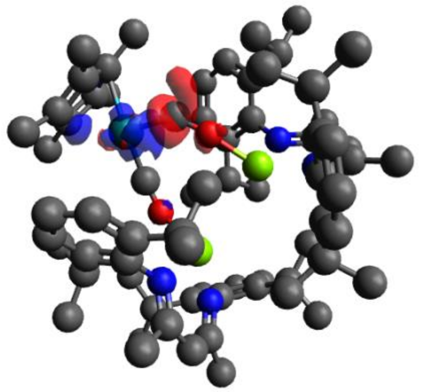

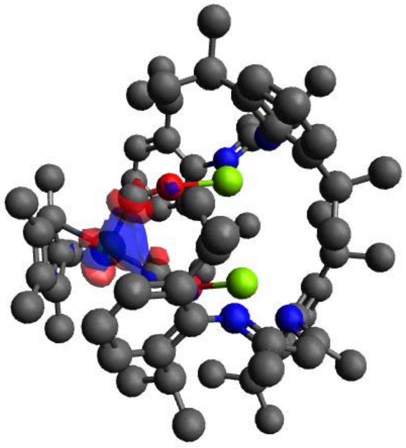

Table S11. Selected deformation density data on complexes **3**. Charge flow from red to blue.

3 ΔE_{ORB}	$\Delta\rho2$
**3a**<br>**-92.2 kcal mol^{-1}**	$\Delta\rho2$<br>Cr (3d) to C (p)<br>-11.7 kcal mol^{-1}
**3b**<br>**-191.7 kcal mol^{-1}**	$\Delta\rho2$<br>W (5d) to C (p)<br>-11.7 kcal mol^{-1}
**3c**<br>**-115.5 kcal mol^{-1}**	$\Delta\rho2$<br>Fe (3d) to C (p)<br>-14.1 kcal mol^{-1}

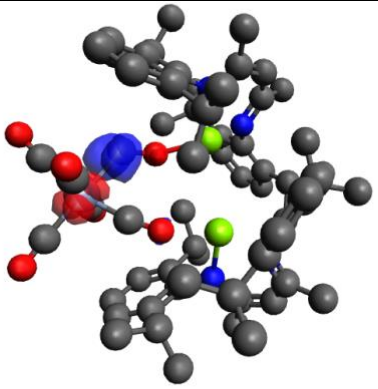

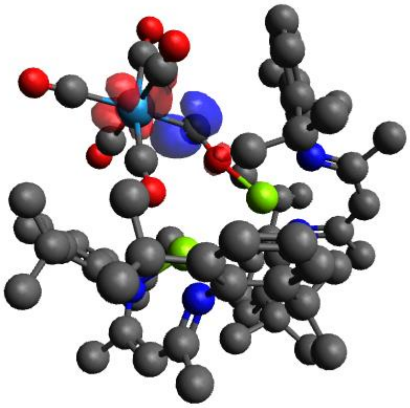

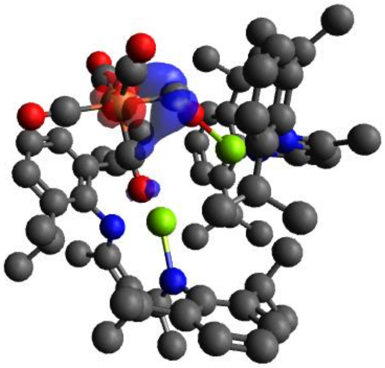

3d
-107.6 kcal mol^{-1}

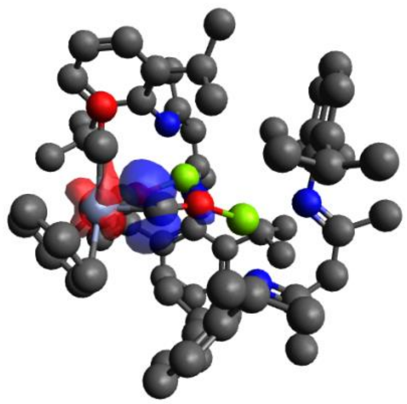

Δρ2
Cr (3d) to C (p)
-18.8 kcal mol^{-1}

3e
-107.7 kcal mol^{-1}

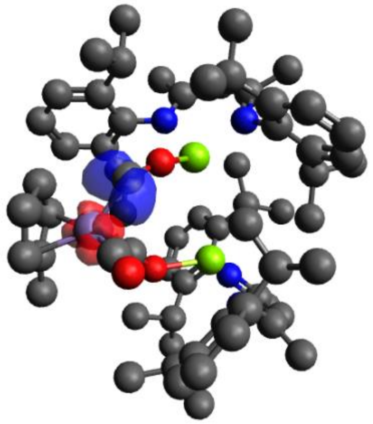

Δρ2
Mn (3d) to C (p)
-16.5 kcal mol^{-1}

3f
-108.6 kcal mol^{-1}

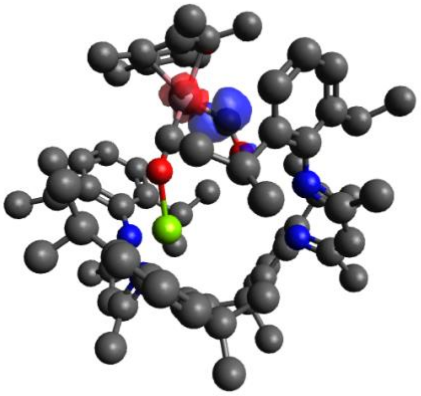

Δρ2
Co (3d) to C (p)
- 23.8 kcal mol^{-1}

3g
-155.6 kcal mol^{-1}

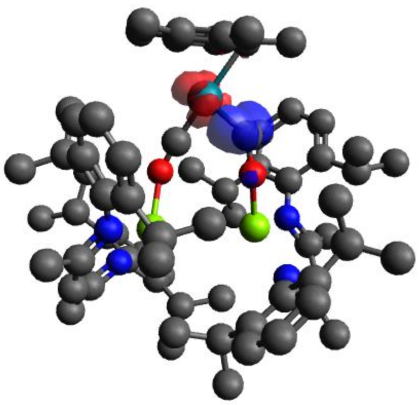

Δρ2
Rh (4d) to C (p)
-22.9 kcal mol^{-1}

3h
-579.1 kcal mol^{-1}

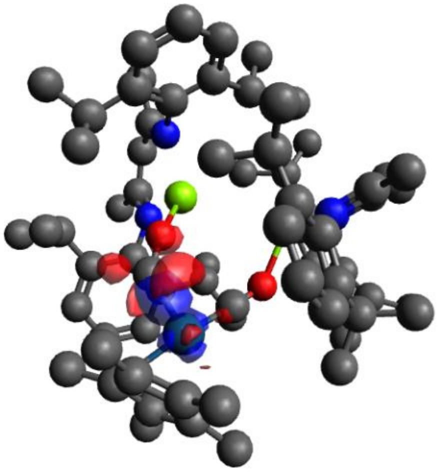

Δρ2
Ir (5d) to C (p)
-82.2 kcal mol^{-1}

Table S12. Selected deformation density data on complexes **3**. Charge flow from red to blue.

<table>
<tr><th>ΔE_{ORB}</th><th>$\Delta\rho 3$</th></tr>
<tr><td>3a
-92.2 kcal mol^{-1}</td><td>
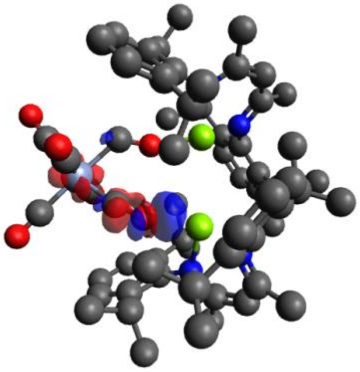

$\Delta\rho 3$
Cr (3d) to C–O (π^*)
-8.3 kcal mol^{-1}</td></tr>
<tr><td>3b
-191.7 kcal mol^{-1}</td><td>
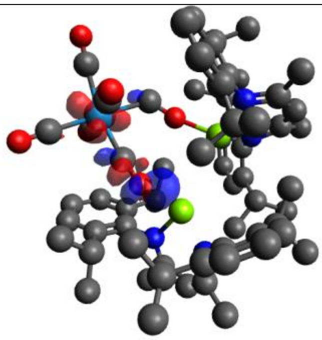

$\Delta\rho 3$
W (5d) to C–O (π^*)
-10.8 kcal mol^{-1}</td></tr>
<tr><td>3c
-115.5 kcal mol^{-1}</td><td>
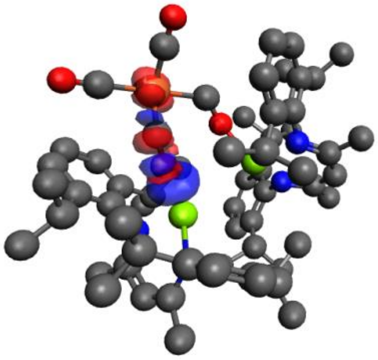

$\Delta\rho 3$
Fe (3d) to C–O (π^*)
-10.3 kcal mol^{-1}</td></tr>
</table>

3d
-107.6 kcal mol^{-1}

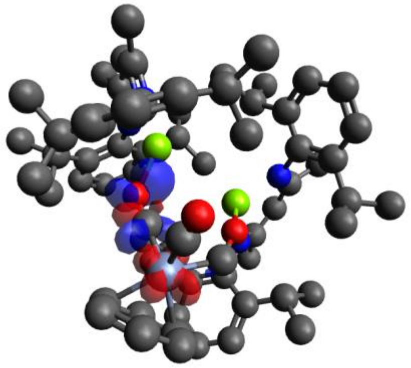

Δρ3
Cr (3d) to C−O (π*)
-12.8 kcal mol^{-1}

3e
-107.7 kcal mol^{-1}

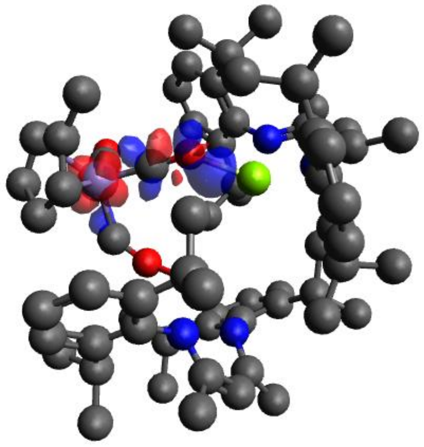

Δρ3
Mn (3d) to C−O (π*)
-12.9 kcal mol^{-1}

3f
-108.6 kcal mol^{-1}

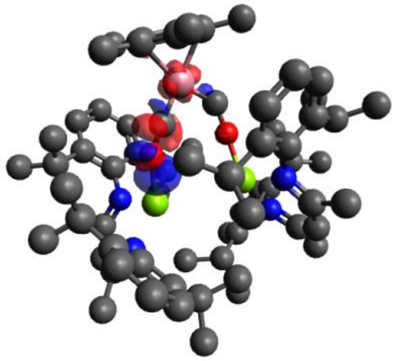

Δρ3
Co (3d) to C−O (π*)
-10.5 kcal mol^{-1}

<table>
<tr><td>3g
-155.6 kcal mol^{-1}</td><td>

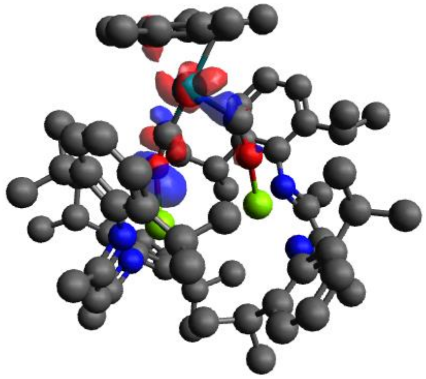

Δρ3
Rh (4d) to C–O (π*)
-12.2 kcal mol^{-1}</td></tr>
<tr><td>3h
-579.1 kcal mol^{-1}</td><td>

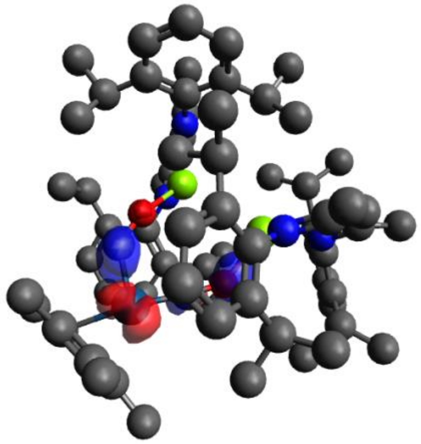

Δρ3
Ir (5d) to C–O (π*)
-12.3 kcal mol^{-1}</td></tr>
</table>

Table S13. Selected deformation density data on complexes **3**. Charge flow from red to blue.

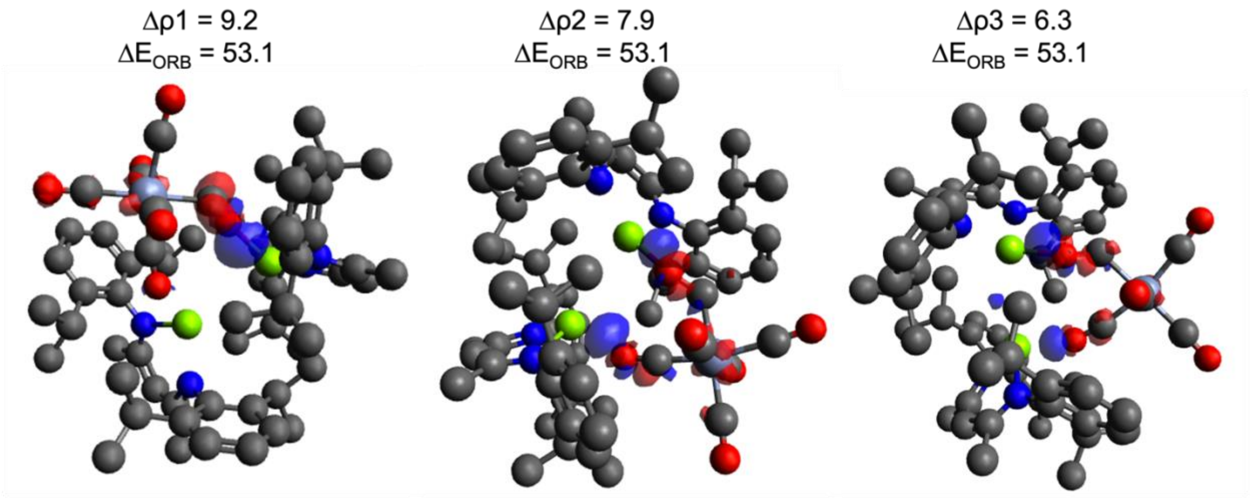

Figure S34. Selected deformation density data on complex **3a** for splitting (ii)- a representative example. All cases **3a-h** show similar ionic interaction between isocarbonly and formyl oxygen atoms and magnesium cations. All values in kcal mol^{-1}. Charge flow from red to blue.

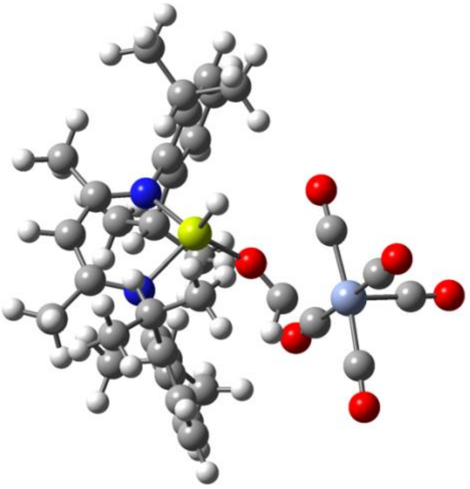

Figure S35. Optimised geometry structure for **Int1**.

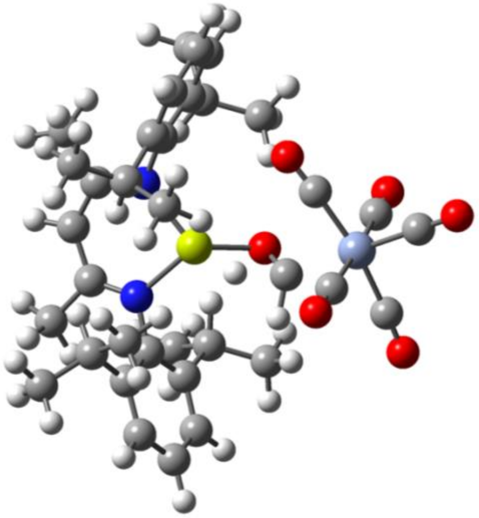

Figure S36. Optimised geometry structure for **TS1**.

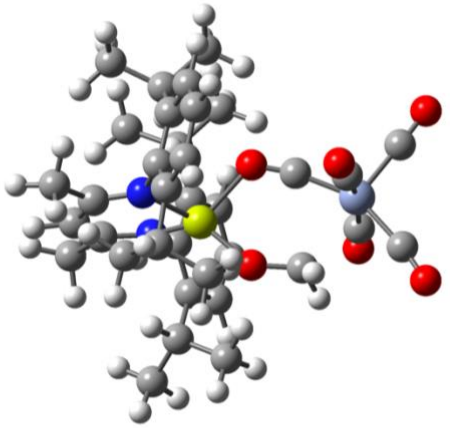

Figure S37. Optimised geometry structure for **Int2**.

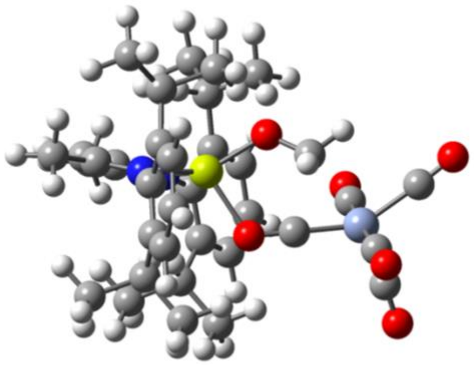

Figure S38. Optimised geometry structure for **TS2**.

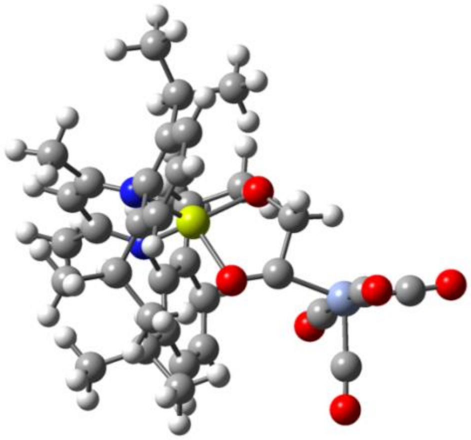

Figure S39. Optimised geometry structure for **Int3**.

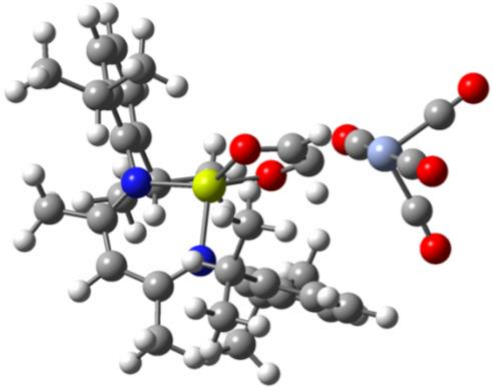

Figure S40. Optimised geometry structure for **TS3**.

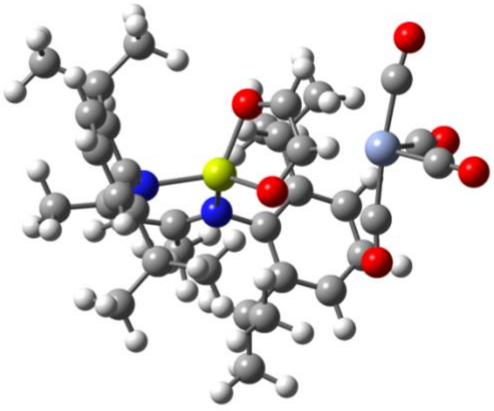

Figure S41. Optimised geometry structure for **Int4**.

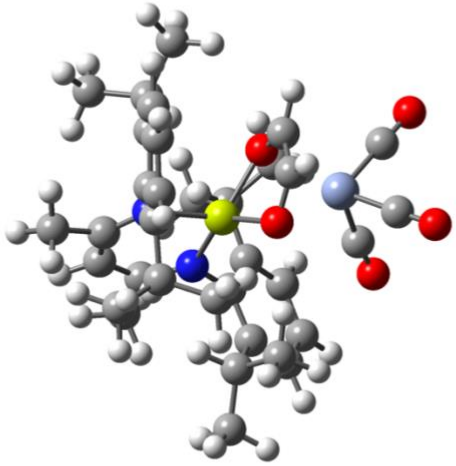

Figure S42. Optimised geometry structure for **4a’**.

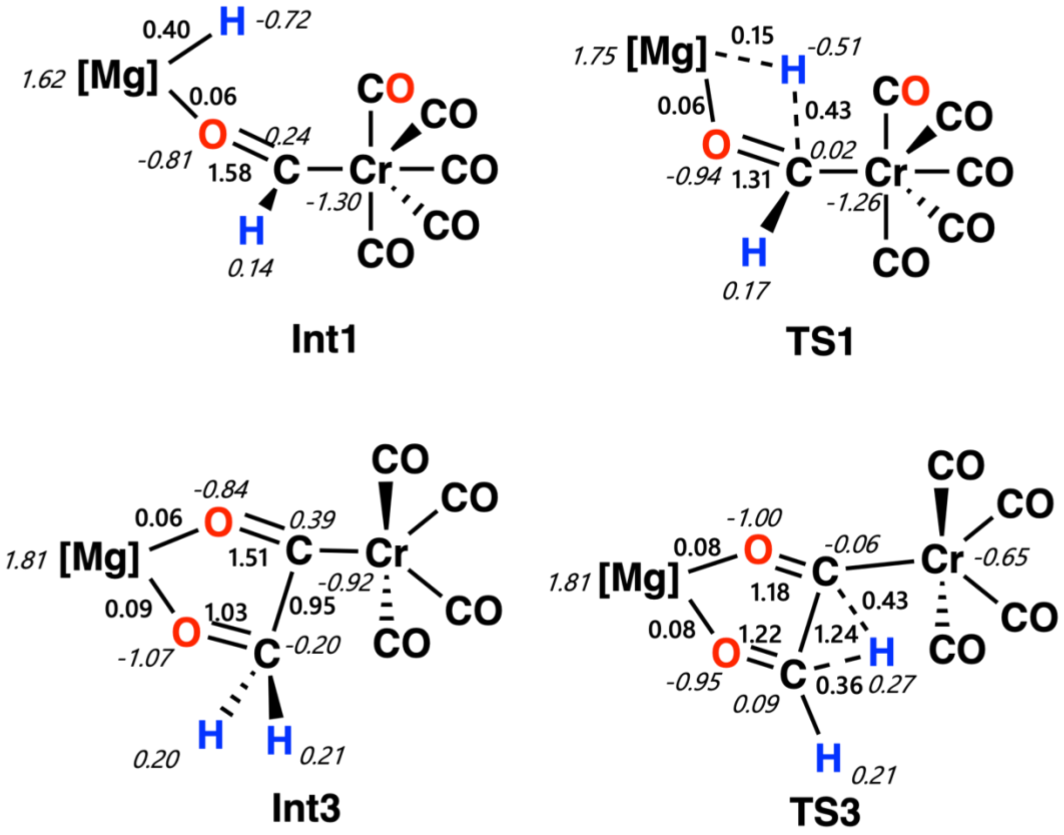

Figure S43. Select *NPA Charges* and **Wiberg Bond Indices** for calculated intermediates and transition states.

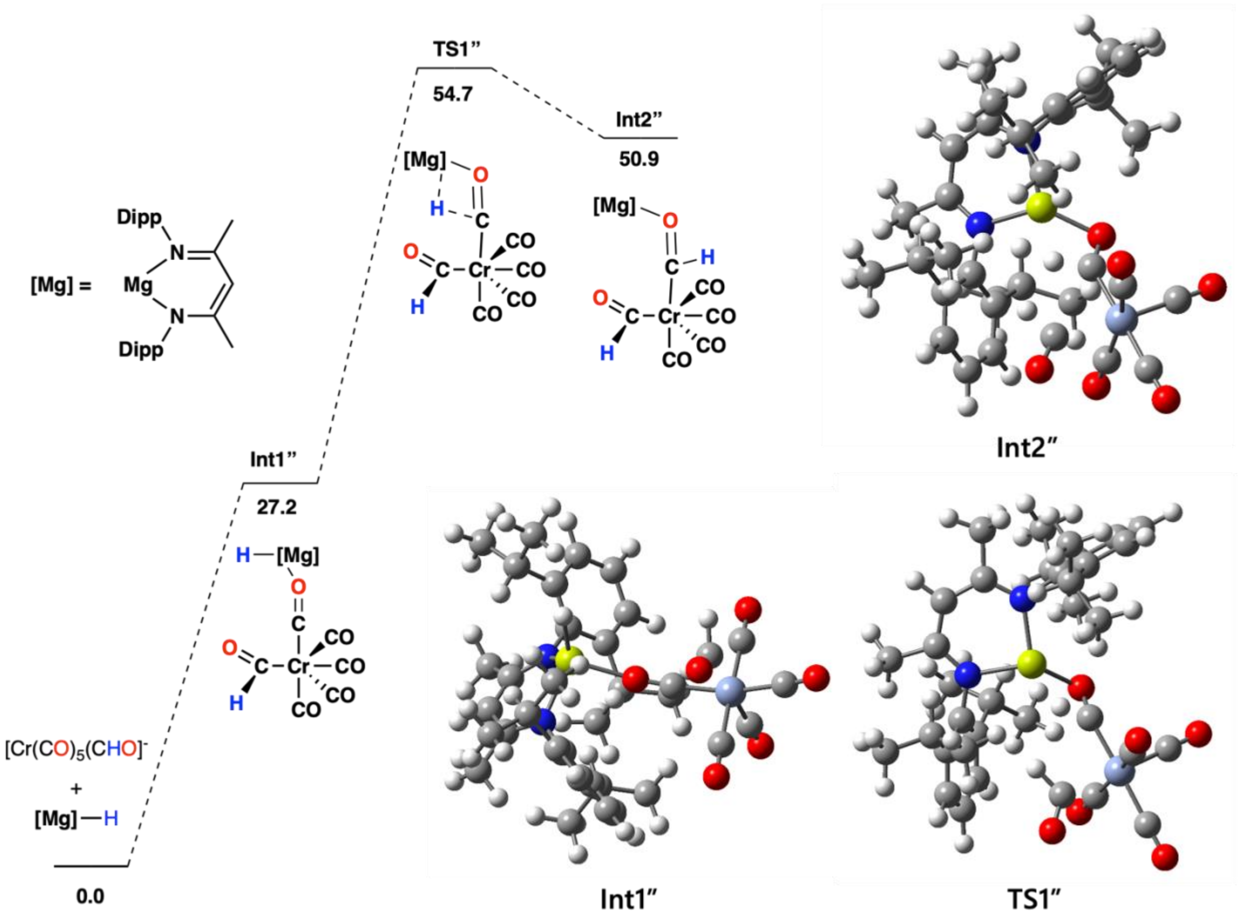

Figure S44. Alternate Potential Energy Surface showing MgH binding to carbonyl ligand *cis* to formyl ligand.

7. References

1 S. P. Green, C. Jones and A. Stasch, *Angew. Chem. Int. Ed.*, 2008, **47**, 9079–9083.

2 S. Kirschner, *Inorganic Syntheses, Volume 23*, 1985.

3 W. K. Wong, W. Tam, C. E. Strouse and J. A. Gladysz, *J. Chem. Soc. Chem. Commun.*, 1979, 530–532.

4 C. P. Casey, M. W. Meszaros, S. M. Neumann, I. G. Cesa and K. J. Haller, *Organometallics*, 1985, **4**, 143–149.

5 B. H. Berke, G. Huttner, O. Scheidsteger and G. Weiler, *Angew. Chem. Int. Ed.*, 1984, 735–736.

6 G. Nelson and C. E. Sumner, *Organometallics*, 1986, **5**, 1983–1990.

7 A. Asdar, C. Lapinte and L. Toupet, *Organometallics*, 1989, **8**, 2708–2717.

8 F. Liang, H. Jacobsen, H. W. Schmalle, T. Fox and H. Berke, *Organometallics*, 2000, **19**, 1950–1962.

9 J. Höck, H. Jacobsen, H. W. Schmalle, G. R. J. Artus, T. Fox, J. I. Amor, F. Bäth and H. Berke, *Organometallics*, 2001, **20**, 1533–1544.

10 F. Liang, H. W. Schmalle, T. Fox and H. Berke, *Organometallics*, 2003, **22**, 3382–3393.

11 Z. Chen, H. W. Schmalle, T. Fox and H. Berke, *Dalton. Trans.*, 2005, 580–587.

12 E. Lu, Y. Chen and X. Leng, *Organometallics*, 2011, **30**, 5433–5441.

13 R. Jana, S. Chakraborty, O. Blacque and H. Berke, *Eur. J. Inorg. Chem.*, 2013, **26**, 4574–4584.

14 G. H. Imler, M. J. Zdilla and B. B. Wayland, *J. Am. Chem. Soc.*, 2014, **136**, 5856–5859.

15 Y. H. Lo, M. C. Li, L. Y. Huang and G. J. Hung, *J. Organomet. Chem.*, 2017, **830**, 109–112.

16 E. S. Wiedner, A. Z. Preston, M. L. Helm and A. M. Appel, *Organometallics*, 2021, **40**, 2039–2050.

17 P. T. Wolczanski, R. S. Threlkel and J. E. Bercaw, *J. Am. Chem. Soc*, 1979, **101**, 218–220.

18 M. R. Churchill and H. J. Wasserman, *J. Chem. Soc. Chem. Commun.*, 1981, **085**, 274–275.

19 L. Li, A. Decken, B. G. Sayer, M. J. Mcglinchey, P. Brégaint, J. Thépot, L. Toupet, J. Hamon and C. Lapinte, *Organometallics*, 1994, **13**, 682–689.

20 B. B. Wayland, B. A. Woods and R. Pierce, *J. Am. Chem. Soc*, 1982, **104**, 302–303.

21 P. T. Barger, B. D. Santarsiero, J. Armantrout and J. E. Bercaw, *J. Am. Chem. Soc*, 1984, **106**, 5178–5186.

22 P. R. Elowe, N. M. West, J. A. Labinger and J. E. Bercaw, *Organometallics*, 2009, **28**, 6218–6227.

23 A. J. M. Miller, J. A. Labinger and J. E. Bercaw, *Organometallics*, 2010, **29**, 4499–4516.

24 Y. Takenaka, T. Shima, J. Baldamus and Z. Hou, *Angew. Chem. Int. Ed.*, 2009, **48**, 7888–7891.

25 J. Cugny, H. W. Schmalle, T. Fox, O. Blacque, M. Alfonso and H. Berke, *Eur. J. Inorg. Chem.*, 2006, **2**, 540–552.

26 M. J. Frisch, G. W. Trucks, H. B. Schlegel, G. E. Scuseria, M. A. Robb, J. R. Cheeseman, G. Scalmani, V. Barone, B. Mennucci and G. A. Peterson, Gaussian 09; Revision D.01, Gaussian Inc.2009.

27 J. Da Chai and M. Head-Gordon, *Phys. Chem. Chem. Phys.*, 2008, **10**, 6615–6620.

28 M. Garçon, C. Bakewell, G. A. Sackman, A. J. P. White, R. I. Cooper, A. J. Edwards and M. R. Crimmin, *Nature*, 2019, **574**, 390–393.

29 M. Garçon, N. W. Mun, A. J. P. White and M. R. Crimmin, *Angew. Chem. Int. Ed.*, 2021, **60**, 6145–6153.

30 M. J. Butler, A. J. P. White and M. R. Crimmin, *Angew. Chem. Int. Ed.*, 2016, **55**, 6951–6953.

31 A. Hicken, A. J. P. White and M. R. Crimmin, *Angew. Chem. Int. Ed.*, 2017, **56**, 15127–15130.

32 J. Tomasi, B. Mennucci and R. Cammi, *Chem. Rev.*, 2005, **105**, 2999–3093.

33 R. Dennington, T. Keith and J. Milliam, GaussView 5.0, Semichem Inc., Shawnee Mission, KS2009.

34 M. P. Mitoraj, A. Michalak and T. Ziegler, *J. Chem. Theory Comput*., 2009, **5**, 962-975.

35 F. Neese, *WIREs Comput Mol Sci* 2018, 8:e1327.

36 F. Neese, F. Wennmohs, U. Becker and C. Riplinger, *J. Chem. Phys.*, 2020, **152**, 1–18.

37 J. Da Chai and M. Head-Gordon, *J. Chem. Phys.*, 2008, **128**, 084106.

38 E. D. Glendening, J. K. Badenhoop, A. E. Reed, J. E. Carpenter, J. A. Bohmann, C. M. Morales, C. R. Landis and F. Weinhold, NBO 6.0. 2013.

39 F. WEINHOLD and C. R. LANDIS, *Chem. Educ. Res. Pr.*, 2001, **2**, 91–104.

40 T. A. Keith, AIMAll (Version 19.10.12). TK Gristmill Software, Overland Park KS, USA, 2019 2019.

41 F. Cortés-Guzmán and R. F. W. Bader, *Coord. Chem. Rev.*, 2005, **249**, 633–662.
